# Highly distinct genetic programs for peripheral nervous system formation in chordates

**DOI:** 10.1186/s12915-022-01355-7

**Published:** 2022-06-27

**Authors:** Rafath Chowdhury, Agnès Roure, Yann le Pétillon, Hélène Mayeur, Vladimir Daric, Sébastien Darras

**Affiliations:** Sorbonne Université, CNRS, Biologie Intégrative des Organismes Marins (BIOM), F-66650 Banyuls-sur-Mer, France

**Keywords:** Ascidian, Amphioxus, Chordates, Peripheral nervous system, EvoDevo, Gene regulatory network, sensory neurons

## Abstract

**Background:**

Vertebrates develop their peripheral nervous system (PNS) from transient unique embryonic structures, the neural crest, and the ectodermal placodes that are located at the border of the forming central nervous system. By contrast, in the invertebrate chordates, amphioxus and ascidians, a large part of the PNS originates at the opposite of the embryo, in the ventral ectoderm. In both groups, a biphasic mechanism regulates ventral PNS formation: high BMP levels specify a neurogenic territory within which glutamatergic epidermal sensory neuron formation is controlled by the Notch pathway. Given these similarities and the phylogenetic relationships within chordates, it is likely that ventral PNS is an ancestral feature in chordates and that it has been lost in vertebrates.

**Results:**

In order to get insights into the molecular control of ventral PNS formation and to test the hypothesis of their homology and potential contribution to the emergence of vertebrate PNS, we undertook a close comparison of ventral PNS formation in the ascidian *Phallusia mammillata* and the amphioxus *Branchiostoma lanceolatum*. Using timed RNA-seq series, we identified novel markers of the ventral PNS during different phases of its development in both species. By extensively determining the expression of paralogous and orthologous genes, we observed that only a minority of genes have a shared expression in the ventral PNS. However, a large fraction of ventral PNS orthologous genes are expressed in the dorsally forming PNS of vertebrates.

**Conclusions:**

Our work has significantly increased the molecular characterization of ventral PNS formation in invertebrate chordates. The low observed conservation of gene expression in the ventral PNS suggests that the amphioxus and ascidian ventral PNS are either not homologous, or alternatively extensive drift has occurred in their regulatory mechanisms following a long period (600 My) of separate evolution and accelerated evolution in the ascidian lineage. The homology to genes expressed in the dorsally forming PNS of vertebrates suggests that ancestral sensory neurons gene networks have been redeployed in vertebrates.

**Supplementary Information:**

The online version contains supplementary material available at 10.1186/s12915-022-01355-7.

## Background

The peripheral nervous system (PNS) allows animals to interact with their environment by receiving external sensory cues. Within the chordates superphylum [1], whose specific body plan notably includes a notochord and a dorsal neural tube, vertebrates have a PNS arising, during early embryonic development, from the frontier between the neural ectoderm and the non-neural ectoderm commonly called the neural plate border (NPB) [2]. The NPB gives rise to two vertebrate-specific dorsal structures: the neural crest and the ectodermal placodes [3, 4]. Several cells constituting these structures have the particularity to delaminate from the dorsal neural tube and then migrate within the embryo in order to reach a specific tissue. This is achieved via an epithelial-to-mesenchymal transition (EMT) for neural crest cells (NCC), which is not the case for migrating placode progenitors (PP) [5]. Subsequently, these multipotent cells differentiate into a wide variety of cell types to establish the structures of their target tissues (i.e. neural crest and placodes do not only give rise to PNS, but also to a variety of cell types: muscle cells, melanocytes, osteoblasts, etc.). At the onset of neurulation, these derivatives are specified through multiple steps finely modulated by specific gene regulatory networks (GRNs) and major signaling pathways such as fibroblast growth factor (FGF), bone morphogenic protein (BMP), Wnt and Notch [6–10]. However, the neural crest GRN shows differences when comparing some specifier and downstream effector gene expressions between the most basal vertebrate group, the cyclostomes, and the gnathostomes [11, 12]. In other bilaterians, *bona fide* NCC or PP do not exist, making them major vertebrate novelties that are allegedly associated with the “New Head” hypothesis explaining the emergence of vertebrates [13]. However, extensive comparative data indicate that orthologs of some key transcription factors of the NPB are expressed in an equivalent region, the lateral neural border, in other bilaterians [10, 14, 15]. Moreover, the lateral neural border of these animals gives rise to similar cell types.

Invertebrate chordates comprise cephalochordates and tunicates, the latter being the closest relatives of vertebrates [16]. Therefore, with their key phylogenetic positions, these organisms may help us to better understand the evolution of PNS formation within the chordates superphylum [1]. The PNS of cephalochordates and tunicates is composed of epidermal sensory neurons (ESN) during their larval life [14, 17, 18]. In the Floridian amphioxus, *Branchiostoma floridae*, two types of ESNs have been described [19]. The best studied and numerous cells are the type I that are present along the entire antero-posterior axis. They are thought to be mechano- and/or chemoreceptors. They project axons to the dorsal neural tube along long distances to make synaptic connections with interneurons to ultimately control the locomotor capacity of the larva [20]. Type II receptors are assumed to be chemoreceptors and to make synaptic connections with type I receptors or other neurons [19]. Developmental studies indicate that the NPB cells express some markers found in vertebrates such as *Msx*, *Pax3/7*, *Zic* or *Dlx* but do not seem to differentiate into ESN [2]. Furthermore, other NPB specifiers are expressed in different tissues (e.g. *SoxE* in the oral skeleton) and none of these cells have migratory capacity [21]. These observations suggest that despite the partial conservation of the NPB specification module, it is not the origin of the amphioxus PNS unlike olfactores (tunicates and vertebrates).

Knowledge on tunicate PNS is more extensive and has been best described in the larva of the ascidian *Ciona intestinalis*. Their ESNs are distributed along the antero-posterior (AP) axis and are divided into palp sensory neurons (PN), rostral trunk epidermal neurons (RTEN), apical trunk epidermal neurons (ATEN), caudal epidermal sensory neurons (CESN) and bipolar tail neurons (BTN) in the caudal dorsal epidermis [22]. As in vertebrates, most of the PNS is derived from the NPB and their neurons have migratory (e.g. BTN) or non-migratory (e.g. dorsal CESN) capacity. It appears that the posterior part of the NPB is proto-neural crest [15] while the anterior part is proto-placodal [23]. Cells derived from one of these regions can be transformed into the other sensory derivatives when the GRN specifying them is altered [14]. Together, these results suggest that the anterior and posterior NPB of tunicates share a GRN composed of similar modules, and thus that the common ancestor of olfactores possessed the precursors of the GRN that gave rise to the vertebrate NCC and PP [14].

**Fig. 1:**
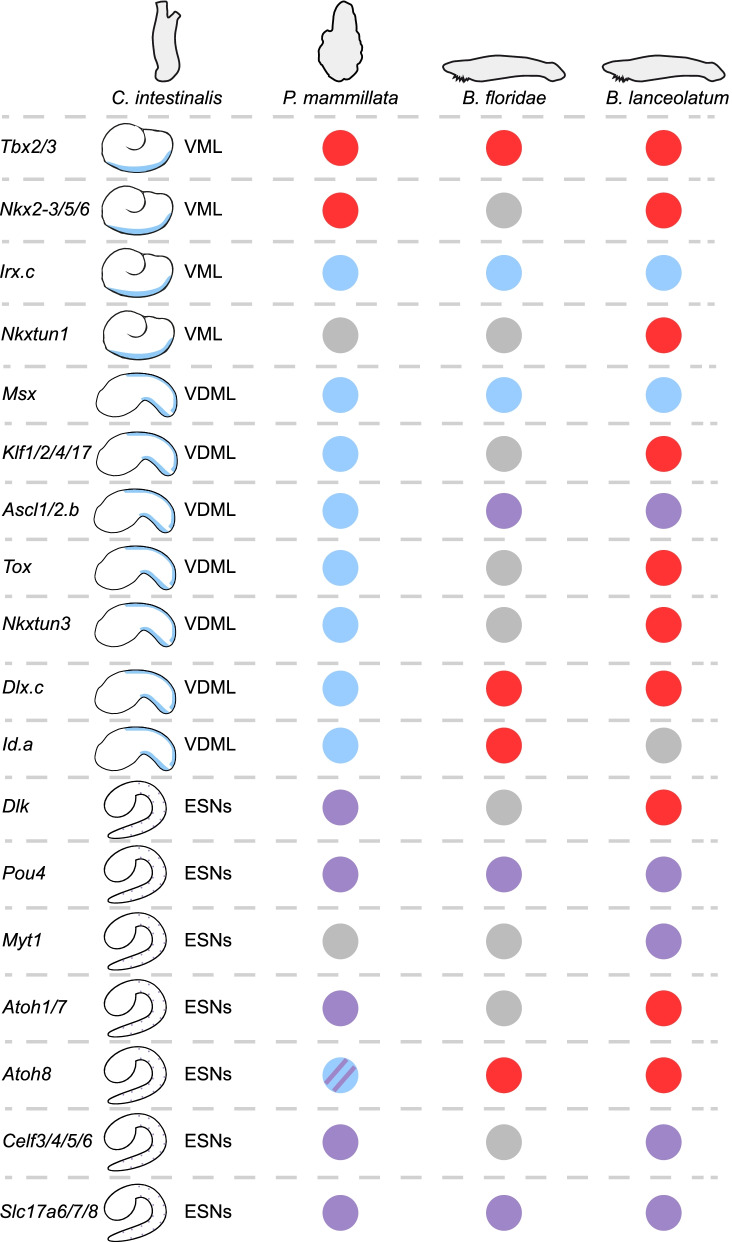
Summary of comparative gene expression of previously known *C. intestinalis* vPNS genes between invertebrate chordates. Results of in situ hybridization are summarized for genes first described in *Ciona intestinalis.* Expression in ventral midline (VML) or both dorsal and ventral midlines (DVML) is represented in light blue in the schematic representation of late gastrula and mid tailbud ascidian embryos. Expression in ESNs is represented by purple dots in the schematic late tailbud ascidian embryos. Expression in the ventral neurogenic field in other species is represented as a light blue circle, expression in ESNs as a purple circle, expression in both as a light blue circle with purple stripes, and lack of expression in vPNS tissues as a red circle. Grey circle: not done. The absence of an ortholog is indicated by a cross

Invertebrate chordates also possess ESNs that are not derived from the NPB, but from the ventral ectoderm [17, 18]; and we will refer to this cell populations as the ventral PNS (vPNS). In the ascidian *C. intestinalis*, the caudal ventral ectoderm is induced as a neurogenic territory at the end of gastrulation by the action of the BMP ligand, Admp [18, 24]. This territory that we will refer to as the ventral midline (VML) is characterized by the early expression of a set of transcription factors (TF): *Tbx2/3*, *Irx.c*, *Nkxtun1* and *Nkx2-3/5/6* [18, 24, 25] (Fig. 1). Later on (from neurula stages), a different set of TFs start to be expressed in the VML: *Msx*, *Nkxtun3*, *Dlx.c*, *Ascl1/2.b* (previously known as *Ascl.b*), *Id.a* (previously known as *Bhlhtun1*), *Klf1/2/4/17* (previously known as *Klf1/2/4*) and *Tox* [18, 24, 26]. Interestingly, these genes are also expressed in the dorsal midline (DML) that derives from the NPB and that is also a neurogenic territory giving rise to dorsal CESNs. A recent study proposes that Msx directly activates the expression of *Klf1/2/4/17* and *Nkxtun3* while *Tox* would be under the control of Ascl1/2.b, suggesting the involvement of two separate modules during the establishment of the neurogenic territory [27]. Within this region, lateral inhibition by future neurons, expressing the Notch ligand Dlk, will prevent adjacent epidermal cells from adopting a neuronal fate, thus avoiding the formation of an excessive number of CESNs [18]. The Notch pathway regulates the expression of pro-neural TFs, expressed both dorsally and ventrally, such as *Atoh1/7* (previously known as *Atonal*) [28], *Pou4* which is known to be involved in sensory cells development in other bilaterians [29] and the zinc finger transcription factor *Myt1* [28]. They ultimately regulate CESN fate acquisition that can be revealed by the expression of the pan-neuronal marker *Celf3/4/5/6* (previously known as *Celf3.a*) [24]. Interestingly, we have recently shown a high degree of conservation of the molecular mechanisms regulating caudal PNS formation within ascidians [27].

Similarly to ascidians, a biphasic model for vPNS specification has been proposed in the amphioxus *Branchiostoma floridae* [17]. First, a ventral neurogenic field marked by the homeobox transcription factor *Tlx* is established by BMP signaling at the end of gastrulation [30]. Then, lateral inhibition by Notch signaling pathway controls ESN number [17]. ESN express various markers such as the Notch ligand *Dll* (previously known as *Delta*), *Ascl1/2.1* (previously known as *Ash*) [17], *Elavl* (previously known as *Hu-elav*) [31], *Pou4* [32], *Ntrk* [33], *Ebf* [34], *Isl* [35], *SoxB1c* [36], *Six1/2* and *Eya* [37]. ESN progenitors that are born ventrally undergo a dorsal migration within the epidermis and differentiate into separate cell populations as revealed by differential gene expression along the antero-posterior axis [36, 38]

The above studies reveal striking similarities in topology and developmental mechanisms during the formation of the vPNS between ascidian and amphioxus. This suggest that these two structures may be homologous, thus making the vPNS an ancestral chordate feature that was lost during the emergence of vertebrates. To test this hypothesis and gain insights into the formation of the PNS and its evolution during chordate diversification, we chose to probe this at the molecular level (gene expression and regulation). Here, we present a detailed side-by-side functional comparative analysis of vPNS formation in invertebrate chordates, using the European amphioxus *Branchiostoma lanceolatum* and the ascidian *Phallusia mammillata*. By precisely uncovering the action of BMP and Notch signaling pathways, we have identified novel vPNS genes using an RNA-seq-based approach and determined their regulation by these pathways in both amphioxus and ascidian. This allowed us to draw provisional GRNs for vPNS formation. Furthermore, a systematic analysis of orthologous gene expression suggests that vPNS GRNs are poorly conserved in invertebrate chordates and that they have undergone extensive drift. Finally, our results suggest that parts of this GRN may have been redeployed during dorsal PNS formation in vertebrates.

## Results

We started our study by the postulate that vPNS are homologous in invertebrate chordates, and we analysed our results in the frame of this hypothesis. However, its validity will be evaluated in the ‘Discussion’ section.

### Dynamic and conservation of previously known actors in vPNS territories

#### Broad conservation of vPNS gene expression within each phylum

**Fig. 2:**
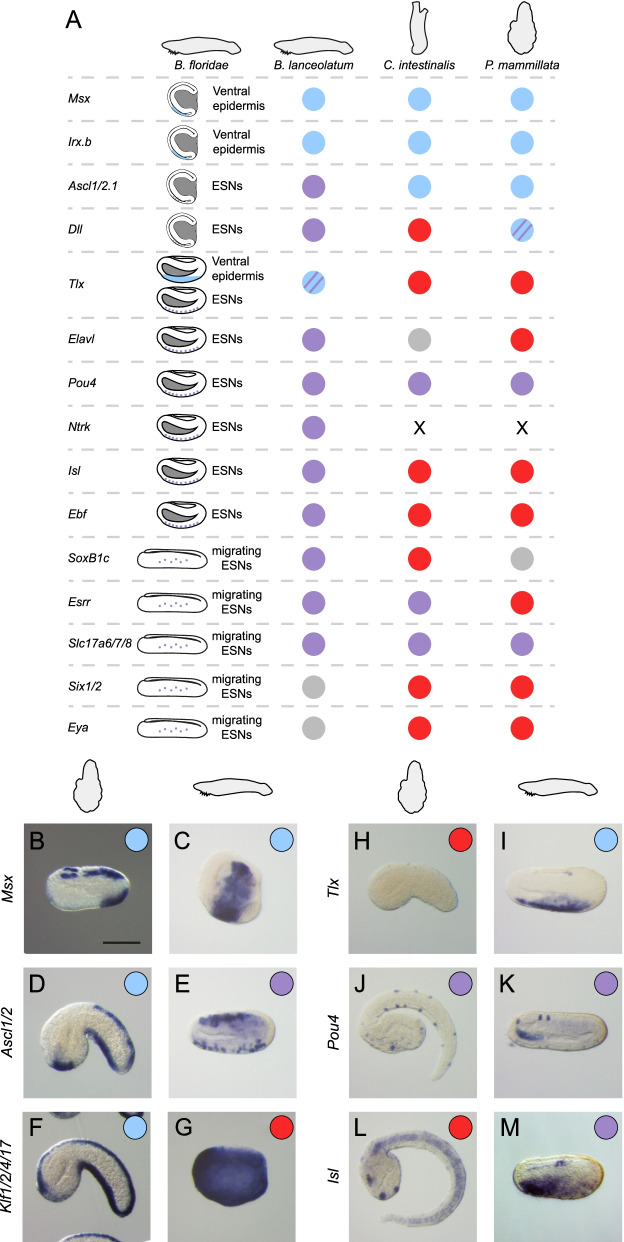
Summary of comparative gene expression of previously known *B. floridae* vPNS genes between invertebrate chordates. **A** Results of in situ hybridization are summarized for genes first described in *Branchiostoma floridae.* Expression in ventral epidermis is represented in light blue in the schematic representation of late gastrula and mid neurula amphioxus embryos. Expression in ESNs is represented by purple dots in the schematic mid neurula and premouth larva amphioxus embryos. Expression in the ventral neurogenic field in other species is represented as a light blue circle, expression in ESNs as a purple circle, expression in both as a light blue circle with purple stripes, and lack of expression in vPNS tissues as a red circle. Grey circle: not done. The absence of ortholog is indicated by a cross. **B–M** In situ hybridization for *Msx* (**B**, **C**) at initial tailbud stage for *P. mammillata* and late gastrula for *B. lanceolatum*, for *Ascl1/2* (**D**, **E**), for *Klf1/2/4/17* (**F**, **G**), for *Tlx* (**H**, **I**) at mid tailbud *P. mammillata* embryos and neurula stages *B. lanceolatum* embryos, for *Pou4* (**J**, **K**) and *Isl* (**L**, **M**) at late tailbud *P. mammillata* embryos and late neurula *B. lanceolatum* embryos. Genes expressed in the ventral neurogenic field are represented by a blue circle and genes expressed in ESNs by a purple circle. Genes with no vPNS-specific expression are represented by a red circle. Embryos are shown in lateral view with anterior to the left and dorsal to the top. Scale bar: 50 μm

As described above, several markers are known to be expressed in the ventral neurogenic field and in the ESNs of the ascidian *C. intestinalis* [18, 24, 26, 28] and in the amphioxus *B. floridae* [17, 39] (Figs. 1 and 2). We first determined whether gene expression was conserved in the model species that we have used here, namely the ascidian *Phallusia mammillata* and the European amphioxus *B. lanceolatum*. In *C. intestinalis*, the dynamic of expression for TFs and signaling molecules in the vPNS is as follows [27] (Fig. 1). During gastrula/neurula stages *Tbx2/3*, *Nkxtun1*, *Irx.c* and *Nkx2-3/5/6* are expressed in the VML but also in the ventral trunk ectoderm where BMP signaling is active [24, 40] with a posterior to anterior dynamic. Then, a new category of genes that are also expressed in the DML start to be expressed, they include *Msx*, *Klf1/2/4/17*, *Ascl1/2.b*, *Tox*, *Nkxtun1*, *Nkxtun3*, *Dlx.c* and *Id.a* [26] (Fig. 1). From tailbud stages, *Dlk*, *Pou4*, *Myt1*, *Atoh1/7*, *Atoh8*, *Celf3/4/5/6* and *Slc17a6/7/8* (also known as *Vglut*) are expressed in both dorsal and ventral ESNs. We have previously shown that several neurogenic midlines and ESNs markers have a conserved expression in *P. mammillata* [27]. Here, we report the expression of additional orthologous genes in *Phallusia* (Fig. 1; Additional file 1: Fig. S1). The ortholog of the ESN marker *Atoh8* was expressed in the ESNs. Interestingly, we could also detect it in the VML at initial tailbud stages in agreement with RNA-seq data for BMP target genes [24]. Surprisingly, we did not observe expression of *Nkx2-3/5/6* and *Tbx2/3* in the VML of *P. mammillata* embryos (only conserved expression in the ventral trunk epidermis was detected) (Additional file 1: Fig. S1). Only *Irx.c* was expressed in the anterior part of the VML, suggesting that early nodes of the vPNS GRN could differ between *Ciona* and *Phallusia*.

In *B. floridae*, the ventral neurogenic field is characterized by the expression of *Tlx* at early neurula stages [17] and precedes later expression of the ESN markers [39]. In the European amphioxus *B. lanceolatum*, we precisely analysed the dynamic of expression of the orthologous genes using time series of embryos. We found a 1 to 1 orthology relationship (Additional file 13: Table S1) and a conserved expression between both amphioxus species (Fig. 2A; Additional file 2: Fig. S2; Additional file 3: Fig. S3) as expected from previous studies [41]. However, we observed differences with what was anticipated from previous studies. The genes *Ascl1/2.1* and *Dll* were expressed in the ventral ectoderm as spots at the end of gastrulation, before the onset of *Tlx* throughout the ventral ectoderm during neurulation. This leads us to propose that the ventral neurogenic field could be established earlier, during gastrula stages, and could be marked by the expression of genes such as *Msx* and *Irx.b* whose expression starts at late gastrula (G4) stage in the ventro-lateral ectoderm (Fig. 2A; Additional file 2: Fig. S2; Additional file 3: Fig. S3). Accordingly, this is a region with active BMP signaling as revealed by P-Smad1/5/8 immunofluorescence (Additional file 4: Fig. S4). Later on, the other ESN markers were expressed at neurula stages, after the initiation of *Tlx* (Fig. 2A; Additional file 2: Fig. S2).

#### Partial conservation of vPNS gene expression between ascidian and amphioxus

We next wanted to know whether ascidian vPNS genes were also expressed in the amphioxus vPNS and vice versa. We thus determined orthology relationships and searched the literature for expression patterns of orthologs/paralogs. When this data was absent, we performed in situ hybridization in *P. mammillata* and *B. lanceolatum* (Additional file 13: Table S1). By merging data from both species, we identified 12 orthogroups with ventral neurogenic territory expression in at least one species. Among them, only 5 had a shared epidermal expression (*Msx*, *Ascl1/2*, *Irx*, *Dlx* and *Klf1/2/4/17*) (Fig. 1; Fig. 2; Sheet 2. in Additional file 13: Table S1). However, *Brlanc.Dlx* and *Brlanc.Klf1/2/4/17* were broadly expressed in the entire epidermis (Additional file 2: Fig. S2); we thus do not consider them having a conserved vPNS expression. The single *Msx* gene was expressed in ventral epidermis in both species. *Irx* has 4 paralogs in *Phallusia* and two of them (*Irx.c* and *Irx.d*) were expressed in ventral epidermis, but only *Irx.c* was expressed in the anterior VML. In *Branchiostoma*, one of the 3 *Irx* paralogs, *Irx.b*, was expressed in the anterior ventral epidermis, suggesting a conserved vPNS expression (Additional file 1: Fig. S1; Additional file 2: Fig. S2). In the case of the midline marker *Phmamm.Ascl1/2.b*, one of its orthologs in *B. lanceolatum*, *Brlanc.Ascl1/2.1*, was not expressed throughout the ventral ectoderm but in isolated ventral ESNs (Fig. 2D, E). Interestingly, we previously described *Ascl1/2* expression in two ascidians that have diverged from *Ciona* and *Phallusia* almost 400 My ago, and its expression was detected in a subset of the VML only, possibly more similar to the expression in amphioxus [27].

We next determined the expression of the orthologs of ESN markers (Fig. 1; Fig. 2). Four of the seven known ascidian CESN marker were expressed in ventral ESNs in amphioxus: *Pou4* (Fig. 2J, K), *Myt1*, *Slc17a6/7/8* and *Celf3/4/5/6* (Additional file 2: Fig. S2). Reciprocally, four of the twelve known amphioxus ESN markers (*Ascl1/2*, *Dll*, *Pou4* and *Slc17a6/7/8*) had an ortholog expressed in *Phallusia* vPNS, in the ESNs except *Phmamm.Ascl1/2.b* as previously described (Additional file 1: Fig. S1). In summary, 8 of the 28 orthogroups with vPNS expression had a conserved expression in invertebrate chordates (Sheet 2. in Additional file 13: Table S1).

### Regulation of vPNS formation by BMP and Notch pathways

We have previously demonstrated that vPNS formation in *Phallusia* was similarly regulated by BMP and Notch pathways as in *Ciona* [27]. We used pharmacological modulation of BMP and Notch pathways in a time-controlled manner to confirm their implication in vPNS formation in *B. lanceolatum* as in *B. floridae*. First, activating the BMP pathway using recombinant BMP4 protein led to the presence of ectopic ESNs in the flank epidermis as revealed by the expression of *Dll* (Additional file 5: Fig. S5A-E). While early treatments (at cleavage or early gastrula stages) had a major impact on dorso-ventral axis as previously reported [17], treatments starting during gastrulation (mid gastrula or late gastrula stages) allowed the formation of embryos with readily visible axis but with a dramatic increase in the number of ESNs (Additional file 5: Fig. S5D, E). Ectopic vPNS on the flanks of the embryos expressed *Dll*, *Tlx* and *Ntrk* (Additional file 5: Fig. S5; Additional file 6: Fig S6). Reciprocally, treatment with the BMP receptor inhibitor Dorsomorphin led to a loss of vPNS formation. Treatment starting anytime between cleavage stages (6 hpf) and neurula stages (16 hpf) abolished *Dll* expression in the ventral epidermis while dorsal expression in the dorsal nervous system was still present. Interestingly, short treatments during a 2-h time window from late gastrula to early-mid neurula (12 to 16hpf) were sufficient to completely repress the expression of *Dll* in the ventral epidermis (Additional file 5: Fig. S5F-S). These results suggest a prolonged requirement for BMP signaling in vPNS formation, in agreement with P-Smad1/5/8 immunostaining (Additional file 4: Fig. S4). Finally, blocking Notch pathway using the gamma-secretase inhibitor DAPT treatment from gastrula stages (12 hpf) led to a major increase of *Ntrk*-positive ESNs in the ventral epidermis (Additional file 5: Fig. S6I), as previously reported in *B. floridae* [17]. Combining BMP4 and DAPT led to a massive increase of *Ntrk*-positive staining almost throughout the entire epidermis (Additional file 6: Fig. S6J).

Previous work has shown that naïve tail ectoderm precursors, b4.2 blastomeres isolated at the eight-cell stage, could be converted into PNS following recombinant BMP4 protein treatment in *C. intestinalis* [18]. However, in *Phallusia*, similar treatment did not activate the expression of *Klf1/2/4/17* in explants (Additional file 6: Fig. S6A, B) while it did in whole embryos [27]. This observation suggests again that some differences may exist in vPNS specification mechanisms between these two ascidian species. The animal blastomeres that are the ectoderm precursors develop into epidermis when isolated at the eight-cell stage in *B. lanceolatum* [42]. We show here that treatment with recombinant BMP4 protein starting at mid gastrula stage was sufficient to induce vPNS fate as revealed by the expression of *Tlx*, *Dll*, *Ascl1/2.1* and *Ntrk* (Additional file 6: Fig. S6E, F, K-R). Combining DAPT induced a dramatic increase in ESN number (Additional file 6: Fig. S6N). These observations demonstrate the possibility to generate almost pure vPNS samples.

### Identification of novel vPNS candidate genes by differential gene expression

**Fig. 3:**
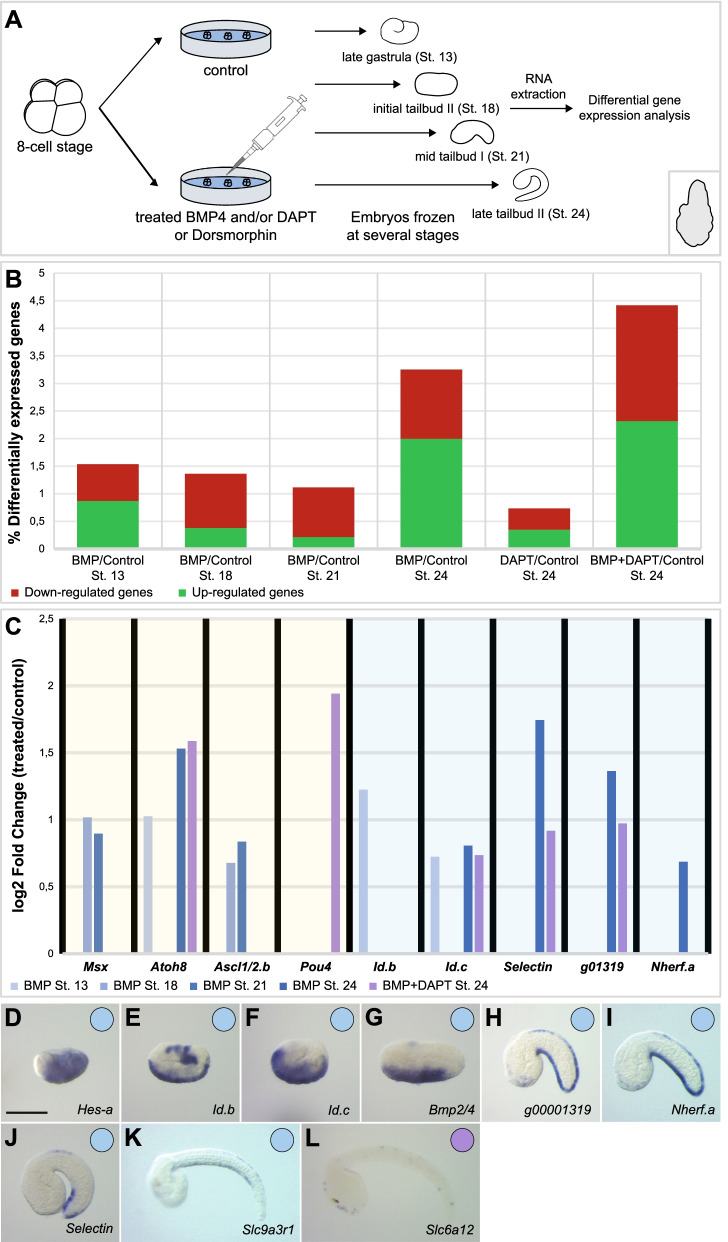
*P. mammillata* vPNS candidate genes identification by RNA-seq analysis on whole embryos. **A** Schematic representation of the experimental design and treatments for DGE analysis at late gastrula, initial tailbud II, mid tailbud I and late tailbud II stages. **B** Quantification of significantly (*p*-value <0.05 calculated by DESeq2) differentially expressed genes (in percentage of the total gene number which is 19,467 genes) from DGE analysis. For each RNA-seq experiment, upregulated genes are indicated in green and downregulated genes in red in the histograms. **C** Differential gene expression of vPNS genes. Log2 fold change of known (yellow backdrop) or candidate (blue backdrop) vPNS genes are shown for samples treated with BMP4 (light blue to dark blue histograms for early to late stages respectively) or BMP4+DAPT (purple histograms for late tailbud stages) compared to control levels. **D–L** In situ hybridization at selected developmental stages for *P. mammillata* candidate genes identified from our RNA-seq data and expressed in the vPNS: *Hes.a* (**D**), *Id.b* (**E**), *Id.c* (**F**), *Bmp2/4* (**G**), *Phmamm.g00001319* (**H**), *Nherf.a* (**I**), *Selectin* (**J**), *Slc9a3r1* (**K**) and *Slc6a12* (**L**). Genes expressed in the VML are represented by a blue circle and genes expressed in ventral ESNs by a purple circle. Embryos are shown in lateral view with dorsal to the top and anterior to the left. Scale bar: 50 μm

**Fig. 4:**
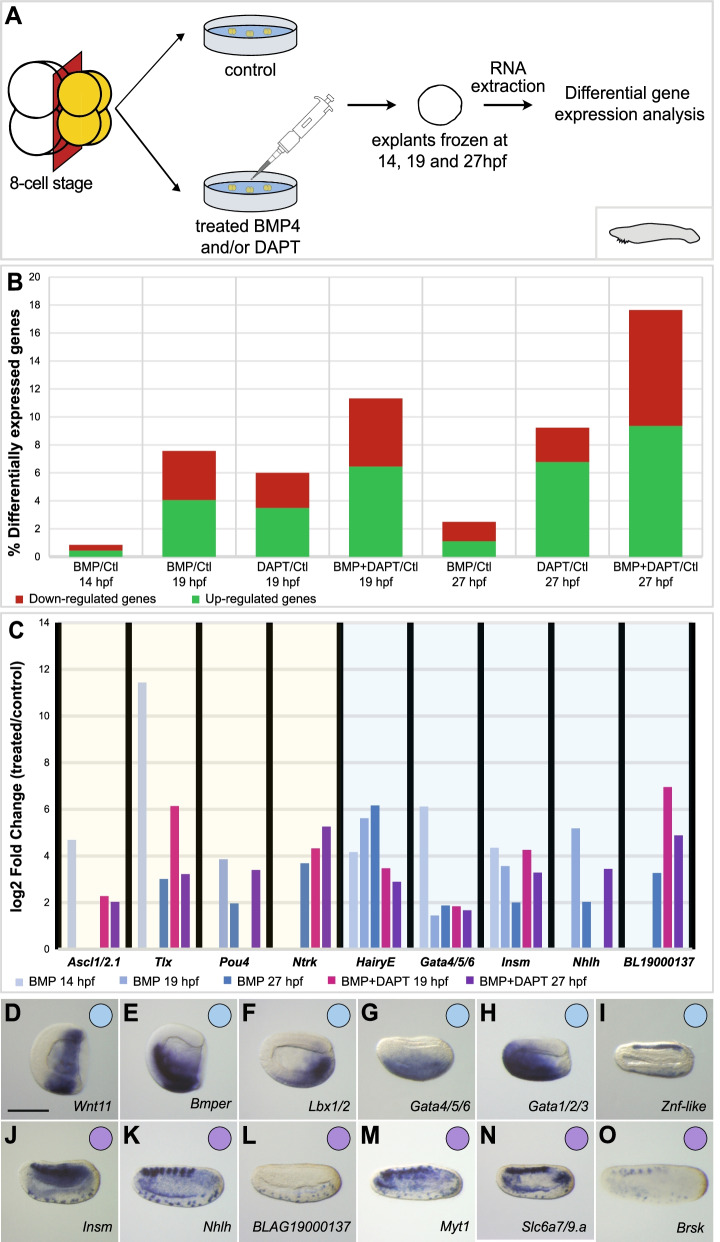
*Branchiostoma lanceolatum* vPNS candidate genes identification by RNA-seq analysis on explants. **A** Schematic representation of animal blastomeres explantation of *B. lanceolatum* and treatments for DGE analysis. Animal blastomeres are indicated in yellow at the eight-cell stage. **B** Quantification of significantly (*p*-value <0.05 calculated by DESeq2) differentially expressed genes (in percentage of the total gene number which is 27,228 genes) from DGE analysis. For each RNA-seq experiment, upregulated genes are indicated in green and downregulated genes in red in the histograms. **C** Differential gene expression of vPNS genes. Log2 fold change of known (yellow backdrop) or candidate (blue backdrop) vPNS genes are shown for samples treated with BMP4 (light blue to dark blue histograms for early to late stages respectively) or BMP4+DAPT (pink and purple histograms for mid and late neurula stages respectively) compared to control levels. **D–O** In situ hybridization at selected developmental stages for *B. lanceolatum* candidate genes identified from our RNA-seq data and expressed in the vPNS: *Wnt11* (**D**), *Bmper* (**E**), *Lbx1/2* (**F**), *Gata4/5/6* (**G**), *Gata1/2/3* (**H**), *Znf-like* (**I**), *Insm* (**K**), *Nhlh* (**K**), *BLAG19000137* (**L**), *Myt1* (**M**), *Slc6a7/9.a* (**N**) and *Brsk* (**O**). Genes expressed in the ventral neurogenic field are represented by a blue circle and genes expressed in ESNs by a purple circle. Embryos are shown in lateral view with dorsal to the top and anterior to the left. Scale bar: 50 μm

To get better insights into vPNS formation and its conservation in invertebrate chordates, we aimed at identifying additional vPNS genes. We thus performed differential gene expression (DGE) analysis by RNA-seq using the above described treatments to modulate BMP and Notch pathways, before determining expression patterns by in situ hybridization for a selection of genes. For *Phallusia*, we used whole embryos at four different stages: late gastrula (St. 13), initial tailbud II (St. 18), mid tailbud I (St. 21) and late tailbud II (St. 24). For the first three stages, we performed BMP4 and Dorsomorphin treatments starting at 8-cell and initial gastrula (St.10) respectively, and for the St. 24, we performed BMP4, DAPT (starting at early neurula stage) and BMP4+DAPT treatments in order to identify neuronal markers (Fig. 3A). In the case of *Branchiostoma*, we produced ectodermal explants. Control explants and explants treated with BMP4 at 8-cell or mid gastrula stage (9 hpf) were frozen at 14, 19 and 27 hpf (equivalent of early, mid and late neurula stages respectively). In addition, explants treated with DAPT and BMP4+DAPT were also frozen at 19 and 27 hpf (Fig. 4A).

In the case of *P. mammillata*, only a few genes were significantly (*p*-value<0.05) differentially expressed in Dorsomorphin versus control conditions, possibly due to the variable effects of the inhibitor depending on the experiment [27]. Therefore, we did not take into account Dorsomorphin conditions when looking at possible vPNS candidate genes. First, we can observe that globally less genes were differentially expressed in *P. mammillata* whole embryos (0.7 to 4.4% of total transcripts differentially expressed, Fig. 3B) than *B. lanceolatum* explants (0.8 to 17.6% of total transcripts differentially expressed, Fig. 4B). However, in both organisms, more genes were differentially expressed at late stages (late tailbud (St. 24) for ascidian and late neurula (27 hpf) for amphioxus) with an even higher number when BMP4 and DAPT treatment were combined. The biological significance of these transcriptomic data was validated by looking at the behaviour of known vPNS genes for each organism (yellow backdrops in Figs. 3C and 4C; Additional file 14: Table S2 and Additional file 15: Table S3). For the ascidian, 13 out of the 15 vPNS genes described above (Sheet 2. in Additional file 14: Table S2) were found to be activated following BMP4 or/and DAPT treatment. Their differential expression generally matched their endogenous expression. The early DVML expressed gene *Msx* was upregulated in tailbud (St. 18 and 21) embryos while the neuronal marker *Pou4* was significantly overexpressed only in BMP4 and DAPT-treated late tailbud (St. 24) embryos (Fig. 3C). For the cephalochordate, 12 out of the 15 vPNS genes were found with the expected deregulation (Sheet 2. in Additional file 15: Table S3). For example, *Tlx* was clearly overexpressed in early neurula (14 hpf) explants when its expression in ventral ectoderm starts in whole embryos, before being overexpressed at later stages in both BMP4 and BMP4+DAPT-treated explants in accordance with its ESN expression. Similarly, the late ESN marker *Ntrk* was only upregulated in late stages explants (Fig. 4C).

Thousands of genes were differentially expressed; therefore, we decided to select genes for expression pattern determination based on a selection of Gene Ontology (GO) terms (Additional file 14: Table S2 and Additional file 15: Table S3). We chose genes upregulated in our temporal RNA-seq data in BMP4 or BMP4+DAPT conditions and, when available, with a GO molecular function associated with TFs or receptors, or a GO biological process associated with the nervous system or cell signaling. In total, 50 and 62 genes were analysed in this study, based on these criteria, for *P. mammillata* and *B. lanceolatum* respectively.

### Expression pattern characterization of vPNS candidate genes

#### Few novel genes expressed in the vPNS in P. mammillata

From the 50 genes studied in *P. mammillata*, only 9 had a clear expression in the vPNS (Fig. 3; Additional file 7: Fig. S7; Additional file 14: Table S2). First, we found two paralogs of the bHLH transcription factor *Id*, *Id.b* and *Id.c*, expressed during gastrulation (St. 13) in the ventral ectoderm. *Id.b* disappeared from the VML at initial tailbud stages (St. 18) before being expressed in both midlines probably excluded from the ESNs starting at mid tailbud stages (St. 21) (Fig. 3E; Additional file 7: Fig. S7). As for *Id.c*, VML expression was maintained in the VML until mid tailbud stage and lost in late tailbud embryo (St. 24) (Fig. 3F, Additional file 7: Fig. S7). For both genes, expression in the CNS and the palps was also detected. The onset of two other genes, the likely tunicate-specific gene *Phmamm.g00001319* and *Nherf.a* (which codes for a scaffolding protein) expression started at mid tailbud in the DVML before being excluded from CESNs at late tailbud (Fig. 3H, I, Additional file 7: Fig. S7). The *Selectin* gene had an expression in the DVML from mid tailbud stage but with a restriction to the posterior caudal region (Fig. 3J, Additional file 7: Fig. S7). The other vPNS genes include *Bmp2/4*, *Hes.a*, *Slc6a12* and *Slc9a3r1* (Fig. 3, Additional file 7: Fig. S7). Interestingly, in our transcriptomic data, we could notice a significant upregulation of these candidate genes concomitantly with their vPNS expression (blue backdrop in Fig. 3C). Thus, *Id.b* was overexpressed only in late gastrulae when it is expressed in the ventral ectoderm whereas *Selectin*, *Phmamm.g00001319* or *Nherf.a* were upregulated at later stages in agreement with their late VML expression.

#### Many vPNS genes in amphioxus

A total of 27 novel genes were expressed in vPNS territories at some point during *B. lanceolatum* embryogenesis (Fig. 4, Additional file 8: Fig. S8; Additional file 15: Table S3), which is more in comparison with *P. mammillata* as expected with the use of explants. As for the previously described vPNS genes, we could categorize three types of expression patterns: 12 genes were expressed in the ventral epidermis, 1 in the ventral epidermis and the ESNs, and 14 in the ESNs only (highlighted in orange in Additional file 15: Table S3). The first category comprises genes like *Gata4/5/6* which was only expressed in the ventral epidermis at early and mid neurula stages before being restricted to the anterior ventral epidermis (Fig. 4G; Additional file 8: Fig. S8), *Lbx1/2* which was expressed in the ventral epidermis at early and mid neurula stages but also in the ventral posterior endoderm (Fig. 4F; Additional file 8: Fig. S8) or the *Znf-like* gene which initiated its expression in the CNS at early neurula stage before being also expressed in the ventral epidermis from mid neurula stage (Fig. 4I; Additional file 8: Fig. S8). In the second group, we find *Wnt11* which was expressed in posterior ventral epidermis at mid gastrula and early neurula stages and had a weak spotted expression on lateral epidermis cells at late neurula stage (Fig. 4D; Additional file 8: Fig. S8). Finally, in the last group, we find genes such as *Insm* or *Nhlh* with an expression in ESNs which initiated most of the time at mid neurula stage. Their expression was, for most of them, also detected in the CNS (Fig. 4J, K; Additional file 8: Fig. S8). During our ISH screening, we identified one gene which seems to be specific to amphioxus (*BLAG19000137*) and expressed exclusively in the ESNs from late neurula stage, similarly to what we observed for *Ntrk* (Fig. 4L, Additional file 8: Fig. S8). Looking at their behaviour in our transcriptomic data, as *Tlx*, we could observe a massive early upregulation for *Gata4/5/6* whereas ESN markers were overexpressed at late stages. Moreover, the profiles of *Nhlh* and *BLAG19000137* were similar to the known ESN markers *Pou4* and *Ntrk* respectively (blue backdrop in Fig. 4C).

In summary, in *P. mammillata*, we identified eight new genes expressed in neurogenic field, but only one CESN marker. For *B. lanceolatum*, several genes were identified for both neurogenic field formation and ESN differentiation/specification. While these genes are expressed in vPNS territories in amphioxus or ascidians, we investigated whether their paralogs and orthologs in the other species were also expressed in these territories.

### Expression pattern characterization of the orthologs and paralogs of newly identified vPNS genes

**Fig. 5:**
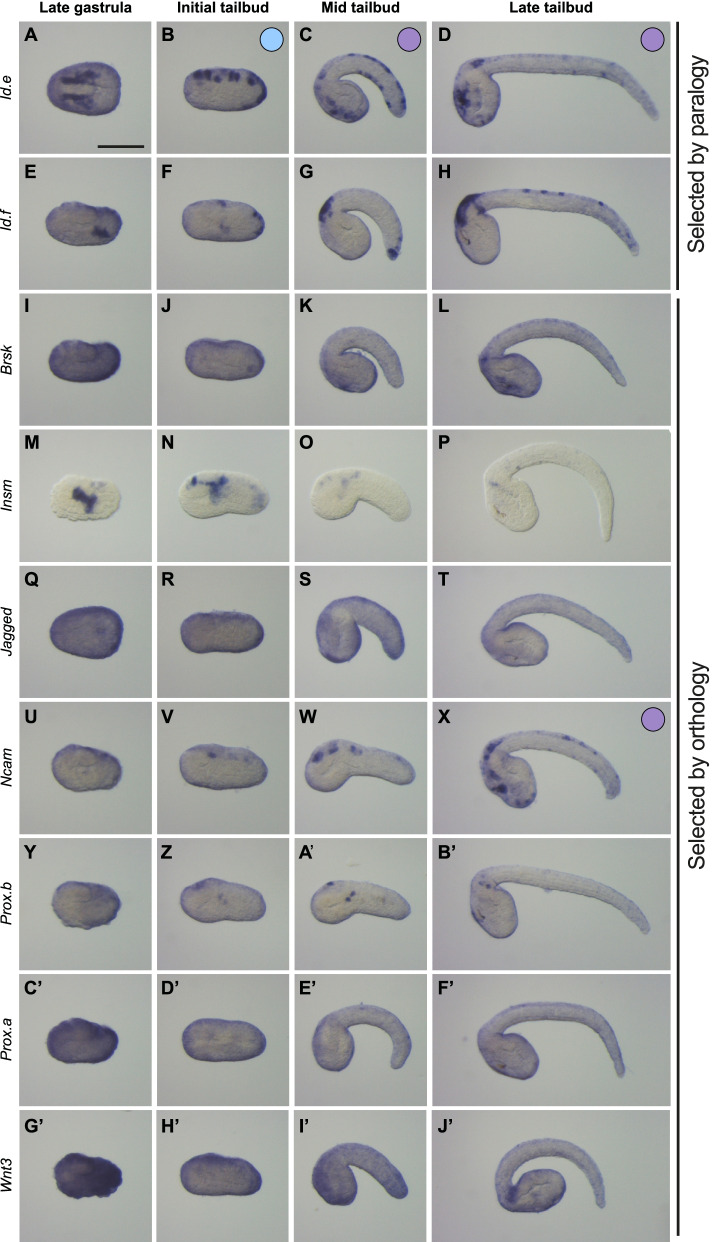
Expression patterns of candidate genes identified by paralogy-orthology analysis in *P. mammillata*. In situ hybridization at several developmental stages for *P. mammillata* candidate genes identified after paralogy analysis with new ascidian vPNS genes (*Id.e* (**A-D**) and *Id.f* (**E–H**)); or orthology analysis with candidate genes identified in amphioxus by RNA-seq: *Brsk* (**I–L**), *Insm* (**M–P**), *Jagged* (**Q–T**), *Ncam* (**U–X**), *Prox.b* (**Y–B’**), *Prox.a* (**C’–F’**) and *Wnt3* (**G’–J’**). Genes expressed in the VML are represented by a blue circle and genes expressed in ventral ESNs by a purple circle. Embryos are shown in lateral view with dorsal to the top and anterior to the left except for **A** and **Q** which are in dorsal view with anterior to the left and right side to the top. Scale bar: 50 μm

We described above the conservation of expression in vPNS territories of the known genes between *P. mammillata* and *B. lanceolatum*. To go further in our comparative analysis in invertebrate chordates, another set of genes has been studied based on paralogy-orthology analysis. We thus performed phylogenetic analysis for several genes (Additional file 12: Data 1), or used phylogenies available in ANISEED (https://www.aniseed.cnrs.fr/), and selected paralogs of newly identified genes or orthologs of new amphioxus vPNS genes in ascidians and vice versa (indicated by “Orthology II” in Additional file 14: Table S2 and Additional file 15: Table S3). For *Phallusia*, eight genes were subsequently analysed. First, we characterized the expression pattern of two other members of the Id family: *Id.e* and *Id.f* that are paralogs of the other *Id* genes described above*. Id.e* was expressed in the posterior ventral epidermis at initial tailbud stage before being expressed in CESNs from mid tailbud stage (Fig. 5A–D). As for *Id.f*, expression was detected in dorsal CESNs in late tailbud embryos (Fig. 5F). Then, the six remaining genes are orthologs of new amphioxus vPNS genes: *Jagged* and *Wnt3* (expressed in posterior ventral epidermis in *B. lanceolatum*); and *Brsk*, *Insm*, *Ncam*, *Prox.a* and *Prox.b* as orthologs of amphioxus ESNs markers. However, we only detected clear expression in the vPNS for the gene encoding for the cell adhesion protein Ncam which was expressed in CESNs from late tailbud stage (Fig. 5X). The transcriptional repressor, *Insm*, was expressed in the mesenchyme and the CNS (Fig. 5M–P), and *Prox.b* in the CNS and trunk lateral cells from late stages (Fig. 5A’,B’). For the other genes, either no expression was detected or there was background staining. Consequently, this paralogy-orthology analysis notably showed that *Ncam* had a conserved expression in ESNs in both species and that 5 of the 6 *Id* paralogs that we identified in the *Phallusia* genome were expressed in the PNS with 4 of them in the vPNS, indicating a possible large implication of this gene family in vPNS establishment in *Phallusia*.

**Fig. 6:**
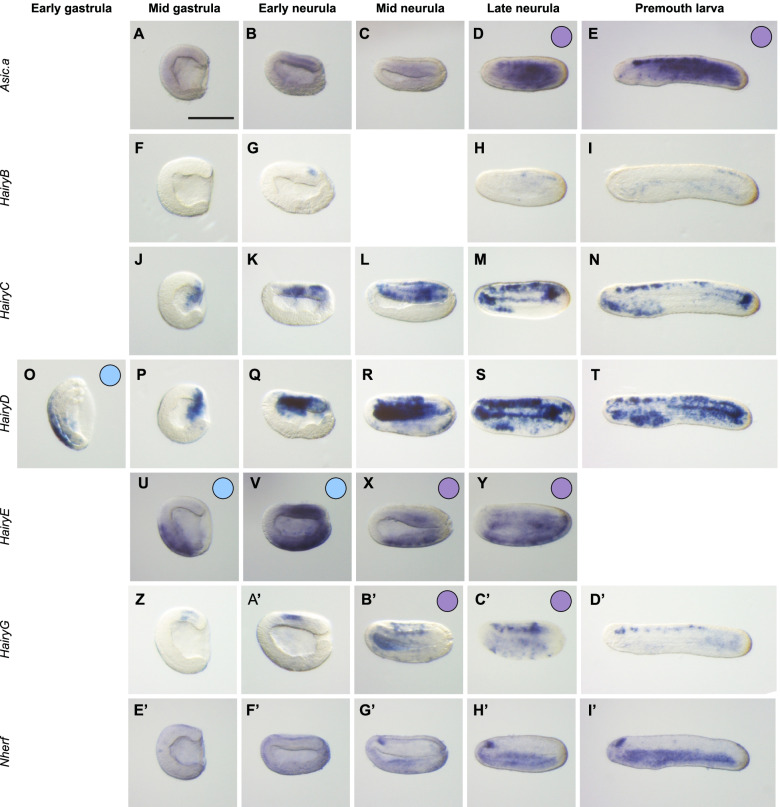
Expression patterns of candidate genes identified by paralogy-orthology analysis in *B. lanceolatum*. In situ hybridization at several developmental stages for *B. lanceolatum* candidate genes identified after paralogy analysis with new amphioxus vPNS gene: *Asic.a* (**A–E**); or orthology analysis with candidate genes identified in ascidian by RNA-seq: *HairyB* (**F–I**), *HairyC* (**J–N**), *HairyD* (**O–T**), *HairyE* (**U–Y**), *HairyG* (**Z–D’**) and *Nherf* (**E’–I’**). Genes expressed in the ventral neurogenic field are represented by a blue circle and genes expressed in ESNs by a purple circle. Embryos are shown in lateral view with dorsal to the top and anterior to the left. Scale bar: 50 μm

In the case of *B. lanceolatum*, we looked at the expression pattern of seven other genes following paralogy-orthology analysis. First, *Asic.a* was expressed in the ESNs at the premouth larva stage (Fig. 6E) as its paralog *Asic.c* (Additional file 8: Fig. S8). The six other genes we have studied are orthologs of *Phmamm.Hes.a*, expressed in the ventral epidermis at the initial gastrula stage (Additional file 7: Fig. S7) or *Phmamm.Nherf.a*. As the phylogeny of the Hairy family is complex, we could not determine a one-to-one orthology; therefore, we looked at 5 *Hairy* genes in amphioxus (Additional file 15: Table S3). Among them, *HairyD* showed an early expression in the ventral epidermis (Fig. 6O), *HairyG* was expressed in ESNs from mid neurula stages (Fig. 6C’) and *HairyE* had an expression in ventral epidermis at mid gastrula and early neurula stages before being expressed in specific ventral cells, possibly ESNs, at mid neurula and late neurula stages (Fig. 6U–Y). The remaining members of the Hairy family (*HairyB* and *HairyC*) did not show expression in vPNS territories (Fig. 6F–N). Finally, *Brlanc.Nherf*, the ortholog of the new DVML marker *Phmamm.Nherf.a*, did not show a conserved expression in the vPNS in amphioxus as it was expressed in the cerebral vesicle from mid neurula stage and in the ventral endoderm later, possibly the future gut (Fig. 6E’–I’). Thus, 4 of the 7 genes that we identified in this paralogy-orthology analysis showed an expression in vPNS territories. Looking at our transcriptomic data, only *HairyE* was upregulated in BMP4-treated conditions whereas the others where downregulated (Additional file 15: Table S3), indicating that our initial approach based only on upregulated genes in our temporal RNA-seq data may contain false negative genes.

### Several new vPNS genes are regulated by BMP and Notch pathways

**Fig. 7:**
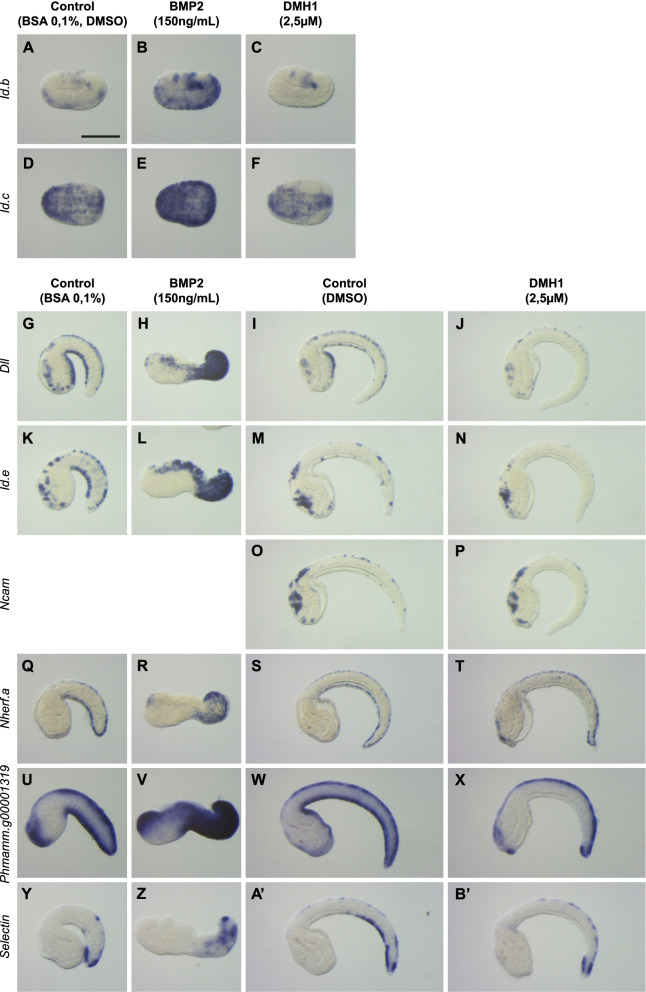
New vPNS genes are regulated by BMP signaling in ventral ectoderm of *P. mammillata* embryos. **A–F** Expression *Id.b* and *Id.c* at early neurula stages in control embryos (**A**, **D**), following treatment from initial gastrula stage (St 10) with BMP2 protein (**B**, **E**) or with DMH1 (**C**, **F**). Expression of *Dll*, *Id.e*, *Ncam*, *Nherf.a*, *Phmamm.g00001319* and *Selectin* in control embryos at mid (**G, K, Q, U, Y**) or late tailbud stages (**I, M, O, S, W, A’**), following treatment from the 8-cell stage with BMP2 protein at mid tailbud stages (**H, L, R, V, Z**) or from initial gastrula stage with DMH1 at late tailbud stages (**J, N, P, T, X, B’**). Embryos are shown in lateral view with dorsal to the top and anterior to the left except for (**D–F**) which are in ventral view with anterior to the left and left side to the top. Each experiment has been performed twice. The number of embryos examined for each condition is between 15 and 40, and the pictures shown correspond to the phenotype observed in over 90% of the embryos. Scale bars: 50 μm

Here, we have directly addressed the regulation of the newly identified vPNS genes by BMP (and Notch for amphioxus) pathways. BMP pathway activation using recombinant BMP2 protein (that proved as efficient as BMP4) treatment from early gastrula stages (St 10) led to a robust ectopic expression of *Id.b* in the entire epidermis at late gastrula stage (Fig. 7A, B). Reciprocally, inhibiting BMP pathway using the pharmacological inhibitor DMH1 (which we found to be more effective than Dorsomorphin during the course of this study) from early gastrula stages (St 10) led to a specific loss of *Id.b* in the ventral epidermis without affecting dorsal expression at late gastrula (Fig. 7C). Similar effects after BMP2 treatment were observed for *Id.c* at late gastrula with its ectopic expression in the whole epidermis (Fig. 7E). However, DMH1 treatment led only to a downregulation but not to a loss of expression (Fig. 7F). In mid tailbud stages, treatment with BMP2 from eight-cell stage led to an ectopic expression of *Dll*, *Id.e*, *Nherf.a*, *Phmamm.g00001319* and *Selectin* (Fig. 7H, L, R, V, Z) in the entire tail epidermis. Finally, at late tailbud stages, treatment with DMH1 induced a complete loss of expression for the ventral ESNs markers *Dll*, *Id.e* and *Ncam* (Fig. 7J, N, P) and a loss of expression except in the most posterior part for the DVML genes *Nherf.a*, *Phmamm.g00001319* and *Selectin* (Fig. 7T, X, B’).

**Fig. 8:**
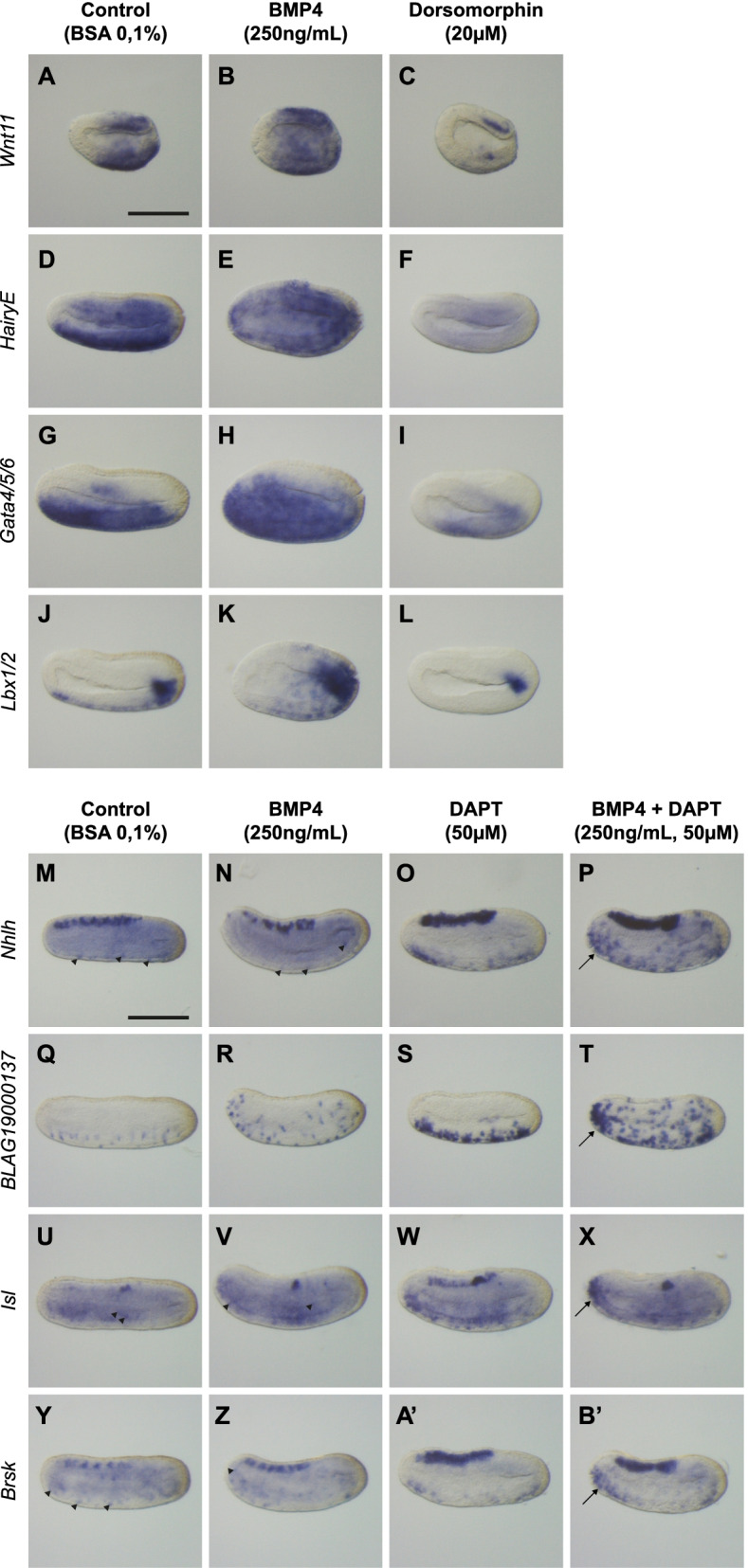
New vPNS genes are regulated by BMP and Notch signaling pathways in *B. lanceolatum* embryos. **A–L** In situ hybridization for candidate genes expressed in ventral epidermis: *Wnt11* at early neurula (**A–C**), *HairyE* (**D–F**), *Gata4/5/6* (**G–I**) and *Lbx1/2* (**J–L**) at mid neurula stages in control embryos (**A, D, G, J**), following treatment from late gastrula stage (12 hpf) with BMP4 protein (**B, E, H, K**) or with dorsomorphin (**C, F, I, L**). **M–B’** In situ hybridization for candidate genes expressed in ESNs: *Nhlh* (**M–P**), *BLAG19000137* (**Q–T**), *Islet* (**U–X**) and *Brsk* (**Y–B’**) at late neurula stages in control embryos (**M, Q, U, Y**), following treatment from late gastrula stage (12 hpf) with BMP4 protein (**N, R, V, Z**), with DAPT (**O, S, W, A’**) or a combination of BMP4 and DAPT (**P, T, X, B’**). Embryos are shown in lateral view with dorsal to the top and anterior to the left. Black arrowheads indicate ESNs when not clearly visible and arrows indicate the anterior most epidermis of the embryo presenting an accumulation of ectopic ESNs. Each experiment has been performed twice. The number of embryos examined for each condition is between 15 and 40, and the pictures shown correspond to the phenotype observed in over 90% of the embryos. Scale bar: 50 μm

In the case of amphioxus, we first analysed the effects of BMP signaling modulation on ventral ectodermal markers expression at two distinct stages (depending on their dynamic of expression): early neurula and mid neurula stages. For most of these markers, activation of the pathway with BMP4 from late gastrula stage led to an ectopic expression in lateral epidermis as observed for *Wnt11* (Fig. 8B), *HairyE* (Fig. 8E), *Gata4/5/6* (Fig. 8H) and *Lbx1/2* (Fig. 8K) whereas treatment with Dorsomorphin induced strong or total repression of their expression in the ventral ectoderm (Fig. 8C, F, I, L). For other markers of this group, we observed unexpected effects such as a weak or absence of repression by Dorsomorphin for *Wnt3* and *Gata1/2/3* or no ectopic expression of *Znf-like* after BMP4 treatment while Dorsomorphin led to its total repression (Additional file 9: Fig. S9). Then, for ESN markers, overactivation of the BMP pathway led to an ectopic expression of ESN in the lateral epidermis as seen for *Nhlh* (Fig. 8N), *BLAG19000137* (Fig. 8R), *Isl* (Fig. 8V) and *Brsk* (Fig. 8Z). DAPT treatment led to an increase of ESNs in the ventral ectoderm (Fig. 8O, S, W, A’) indicating a control of positive cells by Notch pathway as expected. Furthermore, combined treatments of BMP4 and DAPT induced numerous ectopic ESNs in ventro-lateral epidermis (Fig. 8P, T, X, B’). Similarly, four other ESN markers: *HairyD*, *Insm*, *Myt1* and *Prox* were analysed following BMP and Notch pathway alterations and showed the same phenotypes (Additional file 9: Fig. S9). Interestingly, for all these ESN markers (except *HairyD* which was not analysed), we observed that combination of BMP4 and DAPT treatments induced more ectopic ESNs in the anterior most part of the ectoderm.

In summary, for *P. mammillata*, we reported that the 5 new VML markers and the 3 new CESN markers were regulated by BMP signaling in the VML. For *B. lanceolatum*, we analysed 16 new vPNS markers (8 ventral epidermis markers and 8 ESN markers) after alterations of BMP and Notch pathways (for ESNs markers). We showed that 5 out of the 8 ventral epidermis genes we have tackled were expressed ectopically in the lateral epidermis after BMP activation and repressed after BMP inhibition. All of the ESN markers analysed after BMP activation and/or Notch inhibition led to similar effects to previously showed data on known markers. Then, above results suggest that BMP signaling positively regulates most of our new vPNS genes in ascidians and amphioxus, and Notch pathway negatively controls the expression of new ESN markers at least in amphioxus and still need to be tackled in ascidians.

## Discussion

### A molecular atlas of vPNS formation in invertebrate chordates

**Fig. 9:**
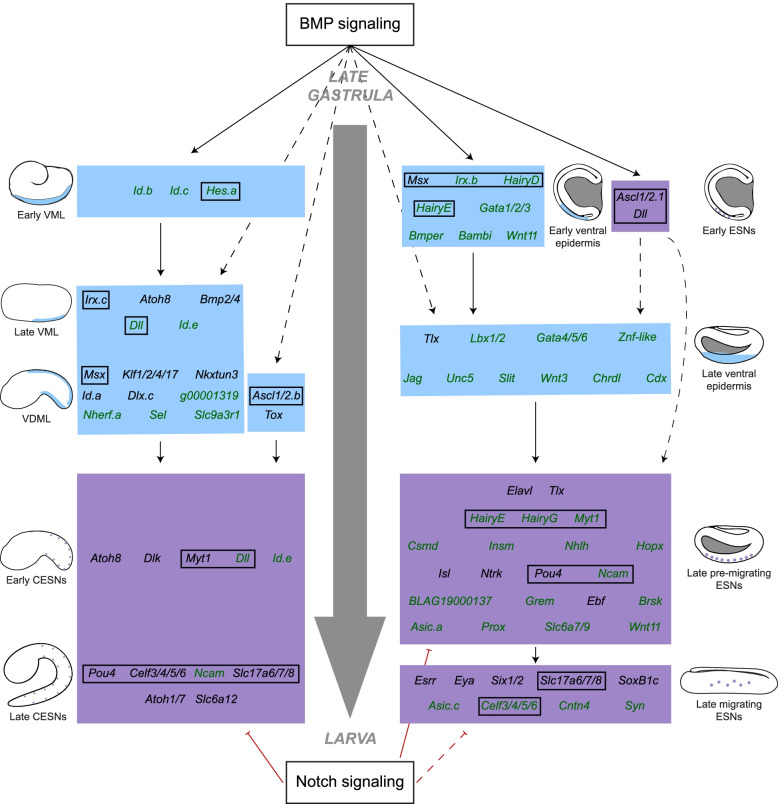
Comparison of hypothetical gene regulatory networks for vPNS formation in invertebrate chordates. The two hypothetical networks were built by combining previously published data together with expression and functional results described in the present study. Embryo diagrams indicate the stage at which the genes initiate their expression in the vPNS and are represented for *Phallusia mammillata* (left GRN) and *Branchiostoma lanceolatum* (right GRN). Light blue backdrop represents genes expressed in the ventral neurogenic field and purple backdrop represents genes expressed in the ESNs. Green font indicates newly identified genes expressed in the vPNS and black boxes indicate orthologs with conserved expression in the vPNS. Solid line indicates known or probable direct regulation of at least one gene of the targeted backdrop while dotted line indicates a hypothetical indirect regulation

Our transcriptomic analysis allowed us to identify 10 and 33 new vPNS markers in *Phallusia mammillata* and *Branchiostoma lanceolatum* respectively (Fig. 9). While we already had a good knowledge on possible GRNs implicated in vPNS formation of ascidians [18, 24, 26, 27], we still identified new genes probably implicated in the ventral neurogenic formation module. Among them, we notably found three other bHLH family member genes: *Id.b*, *Id.c* and *Id.e* that are expressed in ventral neurogenic field and regulated by BMP signaling. Together with the vPNS marker *Id.a*, they are orthologous to the vertebrate *ID3* gene which is implicated in neural crest cell (NCC) formation and regulated by BMP signaling [43]. Then, the likely tunicate-specific gene *Phmamm.g00001319* whose molecular function is not known, and *Nherf.a* require BMP signaling for their VML expression. Their expression patterns suggest that they may negatively regulate ESN formation due to their lack of expression in neuron progenitor cells as observed for other VML markers such as *Ascl1/2.b*, *Id.a* and *Dlx.c* [26]. Finally, two other markers: *Selectin* and *Slc9a3r1*, are expressed later in the DVML and in a restricted pattern along the antero-posterior axis. Thus, the newly identified Id TF genes are probably implicated in gene regulation in the DVML and could give insights into missing links in the previous provisional GRN [27] (Fig. 9). The other genes are more likely involved in differentiation of the ESNs and/or the larval median fin [18]. However, looking at CESN markers, we only identified three new genes expressed in these cells. First, *Dll* and *Id.e* are not only expressed in the VML but also in isolated cells in the DVML, probably CESNs, from early tailbud stages. The last one is the cell adhesion protein-coding gene *Ncam* which is expressed lately in CESNs (Fig. 9). These three genes are also regulated by BMP signaling in the VML.

In amphioxus, in contrast to what was suggested until now, our analysis of the dynamics of vPNS gene expression suggests that the vPNS GRN involves two parallel early modules followed by three sequential late modules (Fig. 9). We looked at early expressed genes (before neurulation) and found seven genes (e.g. *Gata1/2/3*, *Irx.b*) that have an early expression in the ventral ectoderm that requires BMP signaling. Thus, together with *Msx*, they may form an early module for ventral neurogenic field induction in which the first ESN progenitors are specified probably by the action of the pro-neural factor *Ascl1/2.1* and *Dll* (Fig. 9). Then, a second set of genes composed notably of *Tlx*, *Gata4/5/6*, *Irx.b*, *Lbx1/2*, *Znf-like* or *Slit* form a possible late ventral neurogenic field module also modulated by BMP signaling. Interestingly, we observed that during neurulation some of these markers were restricted either anteriorly (e.g. *Irx.b*) or posteriorly (e.g. *Slit*) suggesting a possible antero-posterior patterning of this field. Finally, the two last modules of this hypothetic GRN are composed of several late ESN markers probably implicated in their differentiation before or after their lateral migration (Fig. 9). Among them, we found *Tlx* and *Elavl* (which likely mark all ESN progenitors before their lateral migration) but also several other TFs such as *Pou4*, *Isl*, *Myt1*, *Insm*, *Nhlh* or members of *Hairy* family. Interestingly, within this late pre-migrating ESN module, we observed a possible cascade of temporal expression with (i) some genes that were expressed in the ventral neurogenic field before being detected in isolated neurons (i.e. *Tlx*, *HairyE*), (ii) genes with onset of expression around N2 stage, when ESNs have not started their lateral migration (e.g. *Myt1*, *Insm*, *Nhlh*) and (iii) genes which start their expression later from N4 stages (e.g. *Brsk*, *Isl*, *BLAG19000137*, *Prox*). This last set of genes are often still expressed in migrating ESNs together with other specific markers such as *Celf3/4/5/6*, *Esrr*, *Slc17a6/7/8*, *Synapsin* or *SoxB1c* (Fig. 9)*.* Previous studies proposed that there were probably different populations of ESN coming from the same progenitors, with for example *SoxB1c*-positive ESNs that do not express *Tlx* [36, 39, 44]. Therefore, further studies are necessary to investigate whether the different ESN markers we identified are involved in the differentiation of separate populations.

Finally, our hypothetical GRNs are mostly based on our spatio-temporal gene expression analysis. We still do not know whether the markers that we identified are actual regulators of vPNS formation and how they interact with each other. Thus functional studies such as gene knock-out/knock-down experiments that can be performed using microinjection of morpholinos or CRISPR/Cas9 complexes and that are well established tools in ascidians need to be performed in the future [45–47]. In amphioxus, most of these tools are available for *B. floridae* and *B. belcheri* [48–51], and we are currently establishing the CRISPR/Cas9 approach in *B. lanceolatum*.

### Wnt signaling may be implicated in vPNS formation

When focusing on identifying candidate genes, we noticed the presence of several genes known to be regulated by the Wnt pathway, especially in ascidians. We have previously shown that some members of the ascidian vPNS GRN were modulated by this pathway with the requirement of Wnt for *Msx*, *Klf1/2/4/17* and *Nkxtun3* expression but not for *Ascl1/2.b* [52], indicating that Wnt pathway probably interacts with BMP signaling to regulate one of the two proposed ascidian ventral neurogenic sub-modules (Fig. 9). In addition, we have shown that Wnt signaling represses ESN formation at later stages, indicating that it may also interact with Notch pathway to control ESN number. In amphioxus, *Wnt3* and *Wnt11* were significantly overexpressed in our RNA-seq data following BMP activation. Interestingly, *Wnt11* is expressed in the same territories as *Msx* while *Wnt3* is expressed in the posteriormost epidermis, but both genes are modulated by BMP signaling (Fig. 8B, C; Additional file 9: Fig. S9). These observations suggest that Wnt may also interact with BMP signaling in amphioxus to define the neurogenic field. Following canonical Wnt pathway activation with the pharmacological Gsk3β inhibitor (1-azakenpaullone), we observed that *Tlx* expression was abolished from anterior ventral ectoderm and restricted to the posterior side (Additional file 10: Fig. S10B). Accordingly, the ventral ESN expression of *Elavl* was completely suppressed (Additional file 10: Fig. S10D). Reciprocally, inhibition of the Wnt pathway using the porcupine inhibitor C59 induced an increase in ESN number without obvious effect on the antero-posterior patterning (Additional file 10: Fig. S10F). These preliminary data suggest that Wnt signaling is also involved in ESN formation in amphioxus, and further studies need to be carried out to validate the interaction between these two pathways during vPNS formation.

### Divergent mechanisms regulating vPNS formation in invertebrate chordates

Little is known regarding the homology between ascidian and amphioxus vPNS. On the one hand, both species share similarities such as the existence of ESNs that do not arise from the neural plate border but from the opposite territory, and the ventral ectoderm, and are putative mechanosensory neurons [20, 22] with excitatory glutamatergic synapses [53, 54]. On the other hand, in amphioxus, ESNs undergo epithelial-to-mesenchymal transition (EMT) and migrate dorsally [30] whereas in ascidians these are distinct dorsal peripheral neurons (BTNs) that exhibit this ability [15].

Although our approach was not exhaustive, several conclusions can be drawn regarding genes expressed in vPNS tissues. First, we demonstrate that the two predicted GRNs have only few genes in common, suggesting a weak conservation of the mechanisms underlying vPNS formation in invertebrate chordates (genes in black boxes in Fig. 9). *Msx*, *Irx* and member of *Hairy* family being the genes with conserved ventral epidermis expression in *P. mammillata* and *B. lanceolatum* while *Dll*, *Myt1*, *Pou4*, *Celf3/4/5/6*, *Ncam* and *Slc17a6/7/8* are the shared orthologs expressed in the ESNs. Despite this weak gene conservation, similar regulation by BMP and Notch signaling are observed in invertebrate chordates as observed with our activation and inhibition experiments. Moreover, we have to take into account that our transcriptomics approaches in *P. mammillata* was based on whole embryos and not explants as in amphioxus, thus we may have missed more ascidian GRN candidate genes due to the fact that BMP and Notch signaling are involved in a wide variety of biological processes. However, implications of these signaling pathways are poor indicators of homology since many cells are regulated by them. Indeed, BMP signaling has a major role during dorso-ventral patterning while Notch signaling is often implicated in differentiation between two cell types. In summary, it seems that topologically both vPNS tissues are homologous and composed of glutamatergic epidermal sensory neurons, whereas genetically there is a poor conservation of gene expression.

Finally, the case of *Ascl1/2* is very interesting from an evolutionary point of view. Indeed, in *P. mammillata* we observed that *Ascl1/2.b* is a pan-midline marker whereas in *B. lanceolatum* the ortholog *Ascl1/2.1* is expressed in ESN progenitors suggesting that the function of Ascl1/2 was modified between both species. Interestingly, in a previous study, we showed that *Ascl1/2.b* was expressed in the DVML of Phlebrobranchia (*Ciona* and *Phallusia*) but was an ESN marker in Stolidobranchia (*Molgula* and *Halocynthia*). Moreover, a similar case happens for *Tox*, which allegedly compose one of the two late ventral neurogenic field modules under the regulation of *Ascl1/2.b* (Fig. 9) [27]. Thus, these results lead us to suggest that these two TFs changed their function after the divergence between Phlebrobranchia and Stolidobranchia (390 My). Moreover, the fact that there are two early and late modules in *B. lanceolatum* suggests that this situation may be ancestral in the latest chordate common ancestor and therefore probably in tunicates. Thus, it might be in Phlebrobranchia that this part of the GRN has diverged compared to the ancestral chordate situation. Evidence of developmental system drift also comes from the little degree of conservation for the expression of genes immediately downstream of BMP signaling between *Ciona* and *Phallusia* (Fig. 1; Fig. 9).

Taken together, these observations raise doubts about the hypothesis of homology between the vPNS of ascidians and amphioxus. However, the similarities observed between Stolidobranchia and amphioxus suggest that there may have been an extensive drift in the GRNs leading to the establishment of ascidian vPNS. Then, it would be interesting to perform broad sampling in tunicates (including non-ascidian tunicates), and possibly in other cephalochordate species, to determine whether there is a broader conservation with the proposed GRN of amphioxus and therefore a possible ancestral GRN for the last common ancestor of chordates. Additional approaches, such as *cis*-regulatory module swap between amphioxus and ascidians, may also allow us to better understand the evolution of these two divergent GRNs. Finally, further comparative analysis with other species such as ambulacrarians or vertebrates is needed to tackle more precisely the question of homology of vPNS formation in chordates.

### Similarities between vertebrate dorsal PNS formation and invertebrate chordates vPNS development

While it has been proposed that the GRN involved in dorsal PNS of ascidians is related to GRNs of NCC and PP in vertebrate [14], for the vPNS of ascidians and amphioxus, only limited similarities are observed with vertebrates. In both invertebrate species, we found the expression of transcription factors such as *Msx* in the ventral neurogenic field while it is expressed in the NPB in vertebrate, or the pro-neural transcription factor *Pou4* expressed in the ventral ESNs while being implicated in differentiation of vertebrate mechanosensory neurons [55] and amphioxus ESNs also undergo EMT and migrate dorsally like vertebrate NCCs [30]. Therefore, we aimed at having a global view of the expression of the orthologs of vPNS genes in vertebrates. We could define 58 orthogroups that correspond to 162 genes in mouse and examined their expression during embryonic development using the MGI database (http://www.informatics.jax.org/). For each group, we found at least one ortholog expressed during embryogenesis (Additional file 11: Fig. S11A). Interestingly, over 60% of the genes were expressed in the peripheral nervous system, especially in dorsal root ganglia. However, this approach did not seem to reveal a specific feature of these genes since almost all of them were also expressed in the central nervous system and a large majority in the musculoskeletal system. We undertook a reciprocal approach by examining the proportion of vPNS orthologous genes among the genes expressed in a given tissue (Additional file 11: Fig. S11B). Interestingly, vPNS genes seemed to be enriched in neural crest, ectodermal placodes and enteric nervous system. While this coarse approach will require more careful and detailed examination of expression patterns in several vertebrate species, it is suggestive of similarities between invertebrate vPNS and vertebrate dorsal PNS.

Interestingly, among the multitude of modules known to regulate the specification of NCC and PP [10], one specifying neurons of the sympathetic nervous system (SNS), derived from NCC, bears some similarities with the amphioxus hypothetical GRN. First, in mice it has been shown that BMP and Notch signaling are required for the induction of early sympathetic neuron differentiation and the maintenance of progenitors [56, 57]. Moreover, in this early SNS module, we found several TFs in common with amphioxus vPNS GRN such as *Gata2/3* and *Insm1* which are BMP-induced and essential for sympathetic neuron proliferation and differentiation [8]. Proliferation of SNS is also promoted by *Ascl1* (also called *Mash1*), which is required for the expression of *Insm1* and *Dll1* [58]. The sympathetic nervous system is composed of noradrenergic and cholinergic neurons, and diversification into each type is under the control of two distinct modules. Interestingly, the pro-cholinergic modules also involve orthologs of genes expressed in amphioxus ESN such as *Ntrk3* and *Tlx3*. The LIM-homeodomain transcription factor *Islet1* has also a key role in the SNS development by modulating BMP and Notch pathway and is required for repression of cholinergic fates and maintenance of noradrenergic fates during later developmental stages [57]. However, in the case of amphioxus, ESNs are neither cholinergic nor noradrenergic, but glutamatergic [53].

Other similarities with vertebrate PNS are noticeable. First, a recent study proposed that the Wnt pathway may act in the NCC GRN, notably by regulating early neural crest specifiers such as *MSX1* [59] which is similar to what we observed in ascidians indicating a possible ancestral role of Wnt signaling in chordate PNS formation. Finally, in mice, retinal neurogenesis of the retinal ganglion cells (RGCs) is under the control of a circuit initiated notably by *Ascl1* and *Atoh7* then with downstream factors such as *Islet1*, *Pou4f2*, *Ebf3* or *Myt1* [60]. Together, these observations suggest that the invertebrate chordate modules involved in vPNS development may have been partially recycled dorsally in vertebrates for the differentiation of several sensory cells.

## Conclusions

Our study in invertebrate chordates has increased the molecular description of vPNS formation. Interestingly, a majority of the orthologs of the vPNS genes are expressed in the dorsal PNS of vertebrates. In contrast, for the hypothetical vPNS GRNs of amphioxus and ascidian having few genes in common, we propose that the vPNS is ancestral in chordates and that extensive developmental system drift has occurred since the ancient separation of the two lineages. Future studies focusing on functional experiments would be essential to extend our comparative analysis of these GRNs.

## Methods

### Embryo obtention and manipulation

Adults of *Phallusia mammillata* were collected by diving or during professional trawling in the Banyuls-sur-mer (France) area. Gametes were collected from gonoducts, and egg dechorionation was performed before fertilization as previously described (Darras, 2021) and staging of embryos is according to the developmental table of *Ciona robusta* [61]. *Branchiostoma lanceolatum* adults were collected at the Racou beach in Argelès-sur-Mer (France). Spawning was induced as previously described [62], and staging of embryos is according to Carvalho et al. [63] and by hours post fertilization (hpf) at 19 °C. Explantation experiments on amphioxus were performed using eyelashes as previously described [42].

### Embryos and explant treatments

*Phallusia mammillata* embryos were treated with 150 ng/ml recombinant zebrafish BMP4 protein (1128-BM, R&D Systems Inc, 100 μg/mL stock solution in HCl 4 mM + BSA 0,1%) or recombinant mouse BMP2 protein (355-BM, R&D Systems Inc, 100 μg/mL stock solution in HCl 4 mM + BSA 0,1%) complemented with 0.1% BSA from either the eight-cell stage (St. 4) or initial gastrula stage (St. 10) (both proteins being as potent in our hands), with the BMP receptor inhibitors Dorsomorphin (S7306, Euromedex, 10 mM stock solution in water) and DMH1 (S7146, Euromedex, 10 mM stock solution in DMSO) at 20 and 2.5 μM respectively from eight-cell stage or initial gastrula stage (St. 10), or with 25 μM of the γ-secretase inhibitor DAPT (D5942, Sigma-Aldrich, 10 mM stock solution in DMSO) from early neurula stage (St. 14). For *B. lanceolatum*, embryos and explants were treated with the same molecules but at 250 ng/ml for zebrafish BMP4 and 20 μM for Dorsomorphin. In addition, *B. lanceolatum* embryos were treated with 50 μM of the γ-secretase inhibitor DAPT, 10 μM of the Gsk3β inhibitor 1-azakenpaullone (A3734, Sigma-Aldrich, 10 mM stock solution in DMSO) and 10 μM of the porcupine inhibitor C59 (M3131, Euromedex, 10 mM stock solution in DMSO). Doses and timings of treatments were determined following pilot experiments.

### RNA-seq analysis

Biological samples were frozen in liquid nitrogen: several hundreds of *P. mammillata* control and treated whole embryos at late gastrula (St. 13), initial tailbud (St. 18), mid tailbud (St. 21) and late tailbud (St. 24) stages; 30–60 *B. lanceolatum* control and treated explants at the equivalent of early neurula (14 hpf), mid neurula (19 hpf) and late neurula (27 hpf) stages (except for the 19 hpf samples that were made of 300–400 explants). Each biological sample was produced in triplicates except for the 19 hpf explants samples of *B. lanceolatum* that were produced as duplicates. Total RNA was extracted using the RNeasy Plus Mini Kit (QIAGEN) for whole embryos and the RNeasy Plus Micro Kit (QIAGEN) for explants. Purified RNA quantity and quality was assessed using a Bioanalyzer (2100 Bioanalyzer, Bio2Mar platform, Banyuls-sur-mer). Whole-embryo ascidian samples typically yielded 1–2 μg with a RIN>8.5, while amphioxus explants yielded 100–1000 ng with a RIN>8.5. Library preparation and single-end sequencing were performed either at the Montpellier Genomix platform (MGX, Montpellier, France) with the Illumina HiSeq2500 (single read 50bp) or at the Bio-Environnement platform (LGDP/IHPE, Perpignan, France) with the NexSeq550 (single read 75 bp). Raw reads have been deposited in the NCBI Sequence Read Archive under the accession number PRJNA779382 (https://www.ncbi.nlm.nih.gov/bioproject/779382). Illumina reads were clipped and trimmed to eliminate low-quality regions using Trimmomatic v. 0.38 [64] and then sequence quality was assessed using FastQC v.0.11.7**.** Reads were mapped onto the reference genome assembly MTP2014 available in the ANISEED database (https://www.aniseed.cnrs.fr/) for *P. mammillata* [65] and the unpublished genome assembly braLan3 for *B. lanceolatum* using STAR v.2.7.5.a [66]. Subsequently, read mapping results were summarized in terms of read coverage for genomic features and counted using featureCounts v.1.6.40 [67]. Differential gene expression analysis was performed using SARTools v.1.7.3 [68] and DESeq2 v.2.10.40.6 [69]. Finally, Gene Ontology analysis was performed using the human proteins: the best blast hit Uniprot ID was recovered through blastx against human proteome (proteome ID : UP000005640 in Uniprot database) with a e-value cut-off of 10^−5^, and used in the PANTHER classification system to recover molecular functions and biological processes (http://pantherdb.org/) [70].

### Gene model identifiers, phylogenetic analysis, in situ hybridization and immunostaining

Templates for antisense dig-labelled probes were obtained from cDNA libraries, RT-PCR-amplification or 500 bp synthetic double-stranded DNA (eblock, Integrated DNA Technologies, Leuven, Belgium) based on genomic and/or transcriptomic data (Supplementary Tables S2 and S3). For eblocks, the gene fragment was flanked by T3 and T7 promoter sequences in order to allow amplification by PCR and probe synthesis. Sequences were identified using blast against available transcriptome in ANISEED [65] for *Phallusia mammillata* (using the following assembly: *P. mammillata* MTP2014) and the previously published transcriptome of *B. lanceolatum* [71]. Genes were named using the Uniprot best blast human hit or according to pre-computed orthologies when available in the ANISEED database.

For a limited number of genes (red font in Supplementary Tables S2 and S3), we used blastp against whole proteomes to recover sequences of potential orthologs from *Homo sapiens* (GRCh38.p13, NCBI), *Danio rerio* (GRCz11, NCBI), *Xenopus tropicalis* (UCB_Xtro_10.0, NCBI), *Petromyzon marinus* (kPetMar1.pri, NCBI), *Scyliorhinus canicula* (sScyCan1.1, NCBI), *Ciona intestinalis* (KH, NCBI), *Phallusia mammillata* (MTP2014, Aniseed; and nr from NCBI), *Halocynthia roretzi* (MTP2014, Aniseed), *Molgula occidentalis* (ELv1-2, Aniseed), *Branchiostoma lanceolatum* (braLan3, unpublished), *Branchiostoma belcheri* (Haploidv18h27, NCBI), *Strongylocentrotus purpuratus* (Spur_5.0, NCBI) and *Saccoglossus kowalevskii* (Skow_1.1, NCBI). All sequences were aligned using the MUSCLE program [72]. Maximum-likelihood phylogenies were inferred using PhyML, which determined the best-suited model for each alignment and performed phylogenies with 1000 bootstraps [73]. The resulting trees (Supplementary data 1) allowed us to identify orthologs and paralogs when available, and to infer gene names that may differ from previous publications. We have tentatively followed the nomenclature used in the tunicate community [74].

Whole-mount in situ hybridization were performed as described [52] with Dig-labelled probes synthesized from clones described in Supplementary Tables S2 and S3. Effects on gene expression were analysed for each marker on 15–40 whole embryos or 5–10 explants. Images were acquired using AxioCam ERc5s digital camera mounted on a stereomicroscope (Dicovery V20, Zeiss). Immunofluorescence was performed on samples fixed for in situ hybridization with monoclonal antibodies directed against vertebrate Smad1, Smad5 and Smad9 phosphorylated on 2 serine residues at the C-terminal region (#13820 and #9516, Cell Signaling Technology). The epitope is present in Brlanc.Smad1/5/8 (not shown). The antibodies were used at 1:200–1:800 dilutions followed by detection using an anti-rabbit Alexa488 secondary antibody (A1108, Invitrogen). Both antibodies gave similar results, and results using #9516 are shown in Figure S4. Image acquisition was performed using confocal microscopy (Leica SP8-X, BioPiC platform, Banyuls-sur-mer). Image panels and figures were constructed with Adobe Photoshop and Adobe Illustrator.

## Supplementary Information

**Additional file 1: Fig. S1.** Expression patterns in *P. mammillata* of previously known vPNS markers of invertebrate chordates. *In situ* hybridization at several stages of orthologs of known vPNS markers in invertebrate chordates for *P. mammillata*. Genes expressed in the VML are represented by a blue circle and genes expressed in ventral ESNs by a purple circle. Embryos are shown in lateral view with dorsal to the top and anterior to the left.

**Additional file 2: Fig. S2.** Expression patterns in *B. lanceolatum* of previously known vPNS markers of invertebrate chordates. *In situ* hybridization at several stages of orthologs of known vPNS markers in invertebrate chordates for *B. lanceolatum*. Genes expressed in the VML are represented by a blue circle and genes expressed in ESNs by a purple circle. Embryos are shown in lateral view with dorsal to the top and anterior to the left.

**Additional file 3: Fig. S3**. Dynamical expression of vPNS genes in *B. lanceolatum* embryos. *In situ* hybridization at several stages from early gastrula to premouth larva for the *B. lanceolatum* vPNS genes: *Msx*, *Ascl1/2.1*, *Dll*, *Tlx* and *Elavl*. Light blue frame indicates expression in ventral neurogenic field and purple frame in ESNs. Embryos are shown in lateral view with dorsal to the top and anterior to the left. Scale bars: 50 μm.

**Additional file 4: Fig. S4.** BMP signaling activity during embryogenesis of amphioxus. BMP signaling was detected with an antibody against phosphorylated Smad1/5/8 in *B. lanceolatum* embryos from early gastrula stage (9 hpf) to late neurula stage (24 hpf). DAPI staining shows nuclei of the same embryo for which BMP signaling activity was detected. View of each embryo is indicated in top-left corner of each figure.

**Additional file 5: Fig. S5.** Timing of vPNS sensitivity to BMP activation and inhibition in *B. lanceolatum*. *In situ* hybridization for *Dll* at neurula stages in control embryos (A, F), following continuous treatment with recombinant BMP4 protein starting at various stages (B-E), with dorsomorphin continuously starting at various stages (G-M) or for two hours at the stage indicated on the left of the figure before being extensively washed in seawater (N-S). Embryos are shown in lateral view with dorsal to the top and anterior to the left. Number of embryos analysed and the mean number of ESNs are indicated in the bottom-right corner of the figure. All experiments have been done once except those shown in B and E that have been done twice or more. Scale bar: 50 μm.

**Additional file 6: Fig. S6.** Effects of BMP activation on ectodermal explants of *P. mammillata* and *B. lanceolatum*. Expression of *P. mammillata* midlines marker *Klf1/2/4/17* (A, B) and *B. lanceolatum* ventral ectodermal marker *Tlx* (C-F) and ESNs markers *Ntrk* (G-N), *Ascl1/2.1* (O, P) and *Dll* (Q, R) in whole embryos (C, D, G-J) and ectodermal explants (A, B, E, F, K-R) at several stages. *In situ* hybridization of vPNS genes in control embryos or ectodermal explants (A, C, E, G, K, O, Q), following treatment with BMP4 protein from 8-cell stages (B, D, F, H, L, P, R), with DAPT (I, M) or a combination of BMP4 and DAPT (J, N). The number of whole embryos analysed for each condition is between 15 to 40, and the number of explants is indicated in the bottom-right corner of the figure. All experiments have been done twice except for *Ntrk* and *Ascl1/2.1* that have been done once. Scale bar: 50 μm.

**Additional file 7: Fig. S7.** Other candidate genes expressed in the vPNS of *P. mammillata*. *In situ* hybridization of other *P. mammillata* candidate genes at several stages with newly identified expression in vPNS. Genes expressed in the VML are represented by a blue circle and genes expressed in ventral ESNs by a purple circle. Embryos are shown in lateral view with dorsal to the top and anterior to the left.

**Additional file 8: Fig. S8.** Other candidate genes expressed in vPNS in *B. lanceolatum*. *In situ* hybridization of other *B. lanceolatum* candidate genes at several stages with newly identified expression in vPNS. Genes expressed in the VML are represented by a blue circle and genes expressed in ESNs by a purple circle. Embryos are shown in lateral view with dorsal to the top and anterior to the left.

**Additional file 9: Fig. S9.** Effects of modulating BMP and Notch signaling pathways on new *B. lanceolatum* vPNS genes. **(A-O)** *In situ* hybridization for other vPNS candidate genes at early neurula (A-I) or mid neurula (J-O) stages in control embryos (A, D, G, J, M), following treatment from late gastrula stages with BMP4 protein (B, E, H, K, N) or dorsomorphin (C, F, I, L, O). **(P-A’)** *In situ* hybridization for other vPNS candidate genes at mid (P-W) or late neurula (X-A’) stages in control embryos (P, T, X), following treatment from late gastrula stages with BMP4 protein (Q, U, Y), DAPT (R, V, Z) or combined treatment with BMP4 and DAPT (S, W, A’). Black arrowheads indicate ESN when not clearly visible and arrows indicate the anterior most epidermis of the embryo presenting an accumulation of ectopic ESNs. Embryos are shown in lateral view with dorsal to the top and anterior to the left. Each experiment has been performed twice. Scale bars: 50 μm.

**Additional file 10: Fig. S10.** Wnt signaling modulates vPNS genes expression in *B. lanceolatum*. **(A-D)** *In situ* hybridization for *Tlx* and *Elavl* at mid neurula stages in control embryos (A, C) or following activation of Wnt pathway from late gastrula stages (12 hpf) with 10 μM of 1-azakenpaullone (B, D). **(E, F)** *In situ* hybridization for *Elavl* at mid neurula stages in control embryos (E) or following inhibition of Wnt pathway from late gastrula stages (12 hpf) with 10 μM of C59 (F). Embryos are shown in lateral view with dorsal to the top and anterior to the left. The number of embryos analysed showing the displayed phenotype and, when counted, mean number of ESNs are indicated in the bottom-right corner of the figure. All experiments have been performed once. Scale bar: 50 μm.

**Additional file 11: Fig. S11.** Invertebrate chordates vPNS genes are expressed in PNS territories in mouse. Expression of mouse orthologs were obtained with MGI database (http://www.informatics.jax.org) for all invertebrate chordates vPNS genes (grey), ventral epidermis genes of amphioxus (light blue) and ascidians (dark blue), and ESN of amphioxus (light purple) and ascidians (dark purple). **(A)** Expression of orthologs of invertebrate chordates vPNS genes in several mouse embryonic territories. **(B)** Proportion of mouse orthologs of vPNS genes among the genes expressed in different territories.

**Additional file 12: Data 1**. Phylogenetic analysis. Trees were calculated using maximum likelihood (ML) method with PhyML, and bootstrap supports are given at each node. Further details are indicated in materials and methods.

**Additional file 13: Table S1.** Listing of previously known vPNS genes in invertebrate chordates and their orthologs. Sheet 1. List of previously known vPNS genes in ascidians and their orthologs in amphioxus, and *vice versa*. Highlighting in blue indicates expression in ventral neurogenic field, in purple expression in ESNs and in red expression in other tissues. Highlighting in the gene family name indicates conserved expression in the ventral field (blue), the ESNs (purple), one of them (blue-purple) or non-conserved expression in vPNS (red). Sheet 2. Simplified view of sheet 1. nd: not done.

**Additional file 14: Table S2.** Listing of all *P. mammillata* genes analysed in this study. Sheet 1. List of genes of *P. mammillata* analysed in this study. Red font for gene name indicates that phylogenetic analysis has been carried out. When available, GO term for molecular function (MF) and biological process (BP) are indicated. Highlighting in purple indicates genes coding for transcription factors, blue for receptors, green for genes involved in the nervous system and yellow for genes involved in cell signalling. The origin of template for Dig-probe synthesis (500 bp region for synthetic DNA, cloning primers or clone provenance) is shown. Finally, gene expressed in the vPNS are highlighted in orange. Sheet 2. Extraction from sheet 1 of the *P. mammillata* genes studied by orthology I (known vPNS genes + paralogs + amphioxus vPNS genes orthologs) and expressed in the vPNS.

**Additional file 15: Table S3.** Listing of all *B. lanceolatum* genes analysed in this study. Sheet 1. List of genes of *B. lanceolatum* analysed in this study. Red font for gene name indicates that phylogenetic analysis has been carried out. When available, GO term for molecular function (MF) and biological process (BP) are indicated. Highlighting in purple indicates genes coding for transcription factors, blue for receptors; green for genes involved in the nervous system and yellow for genes involved in cell signalling. The origin of template for Dig-probe synthesis (500 bp region for synthetic DNA, cloning primers or clone provenance) is shown. Finally, genes expressed in the vPNS are highlighted in orange. Sheet 2. Extraction from sheet 1 of the *B. lanceolatum* genes studied by orthology I (known vPNS genes + paralogs + amphioxus vPNS genes orthologs) and expressed in the vPNS.

## Data Availability

All data generated or analysed during this study are included in this published article, its supplementary information files and publicly available repositories. RNA-seq data generated during this study are available under the accession number PRJNA779382 (https://www.ncbi.nlm.nih.gov/bioproject/779382). The ANISEED (https://www.aniseed.cnrs.fr/) and MGI (http://www.informatics.jax.org/) databases were used to examine gene expression during ascidian and mouse embryonic development respectively. Materials are available upon request.

## Abbreviations

ATEN: Apical trunk epidermal neuron
BMP: Bone morphogenic protein
BTN: Bipolar tail neuron
CESN: Caudal epidermal sensory neuron
DGE: Differential gene expression
DML: Dorsal midline
DVML: Dorsal and ventral midlines
EMT: Epithelial-to-mesenchymal transition
ESN: Epidermal sensory neuron
FGF: Fibroblast growth factor
GO: Gene ontology
GRN: Gene regulatory network
NCC: Neural crest cell
NPB: Neural plate border
PN: Palp sensory neuron
PNS: Peripheral nervous system
PP: Placode progenitor
RTEN: Rostral trunk epidermal neuron
SNS: Sympathetic nervous system
TF: Transcription factor
VML: Ventral midline
vPNS: Ventral peripheral nervous system
