# Supplementary figures and images for: Highly distinct genetic programs for peripheral nervous system formation in chordates
Source: BMC Biol. 2022 Jun 27;20:152. doi: 10.1186/s12915-022-01355-7 (PMC9238270; doi:10.1186/s12915-022-01355-7)

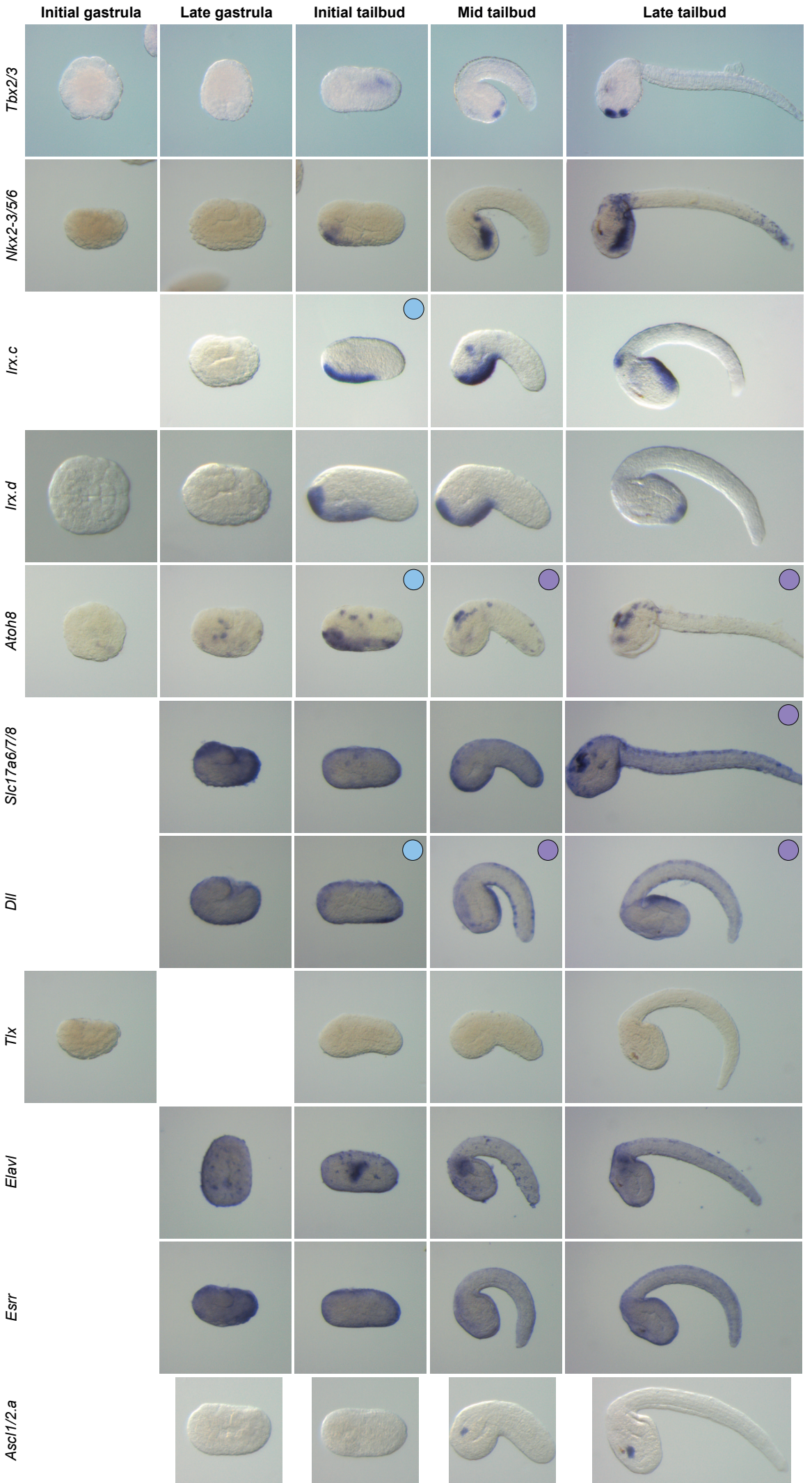

Supplement: Supplementary file 1 — Additional file 1: Fig. S1. Expression patterns in P. mammillata of previously known vPNS markers of invertebrate chordates. In situ hybridization at several stages of orthologs of known vPNS markers in invertebrate chordates for P. mammillata. Genes expressed in the VML are represented by a blue circle and genes expressed in ventral ESNs by a purple circle. Embryos are shown in lateral view with dorsal to the top and anterior to the left. [file 12915_2022_1355_MOESM1_ESM.pdf]

Nkxtun3 orthologs?

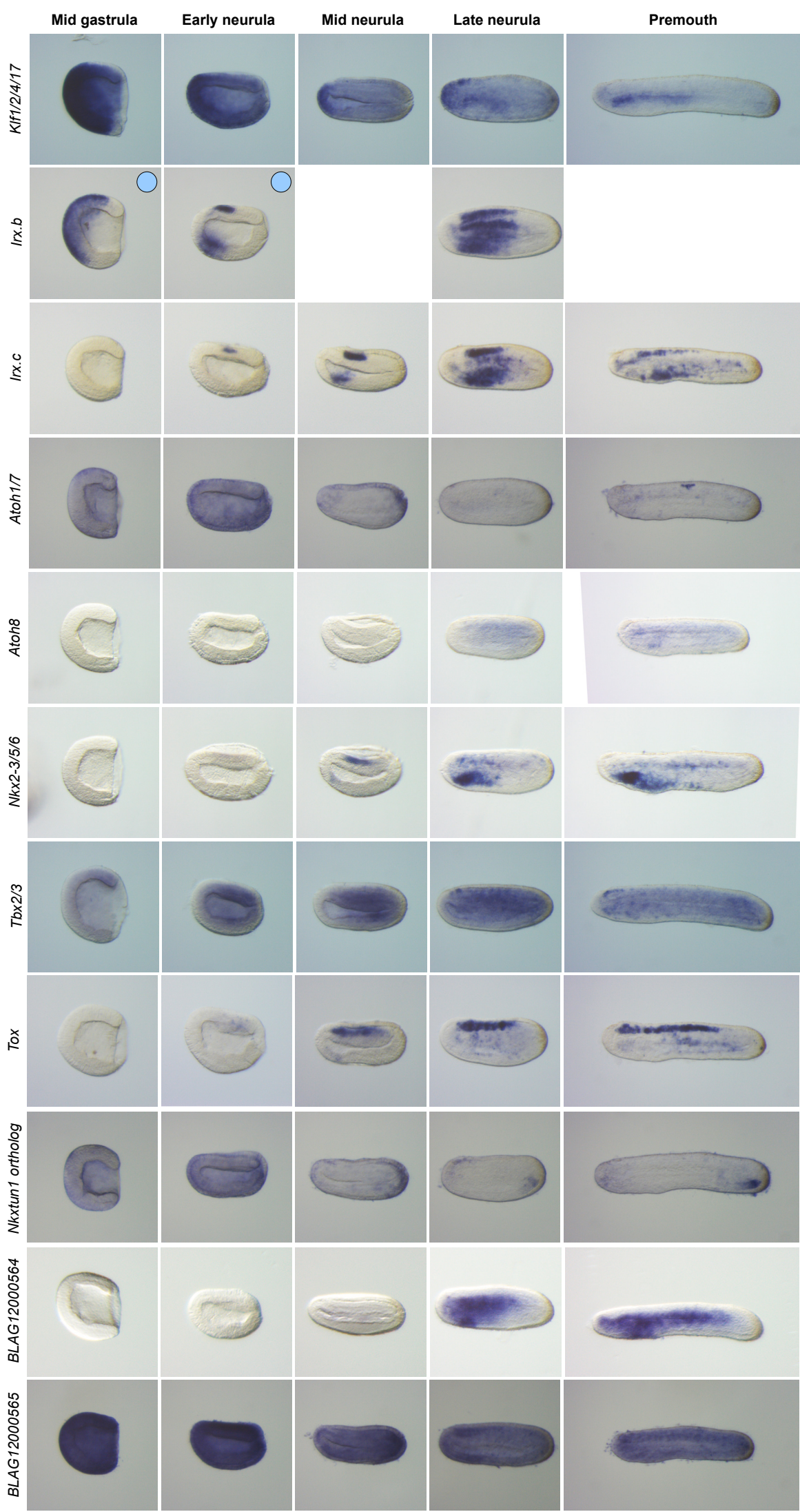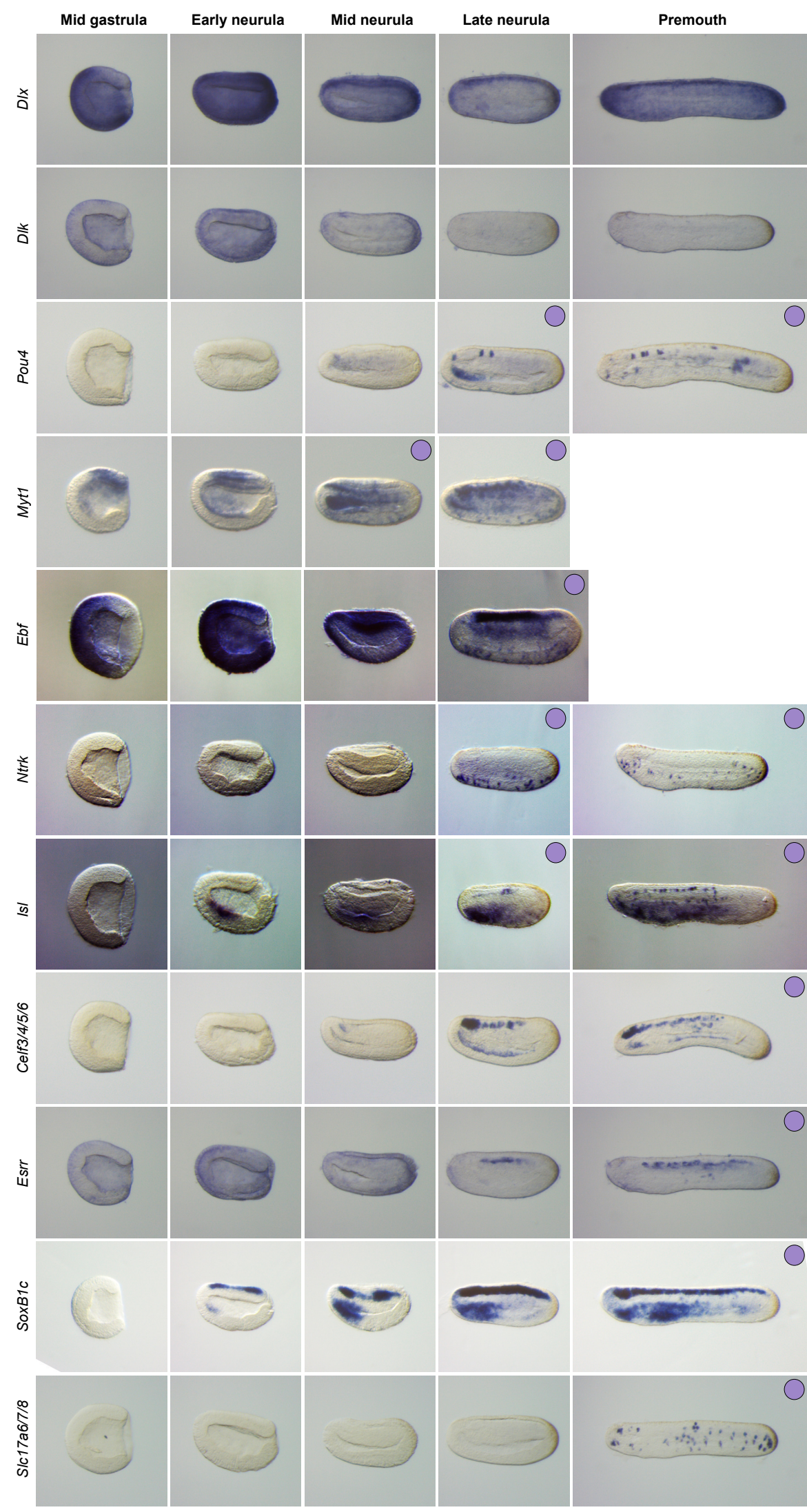

Supplement: Supplementary file 2 — Additional file 2: Fig. S2. Expression patterns in B. lanceolatum of previously known vPNS markers of invertebrate chordates. In situ hybridization at several stages of orthologs of known vPNS markers in invertebrate chordates for B. lanceolatum. Genes expressed in the VML are represented by a blue circle and genes expressed in ESNs by a purple circle. Embryos are shown in lateral view with dorsal to the top and anterior to the left. [file 12915_2022_1355_MOESM2_ESM.pdf]

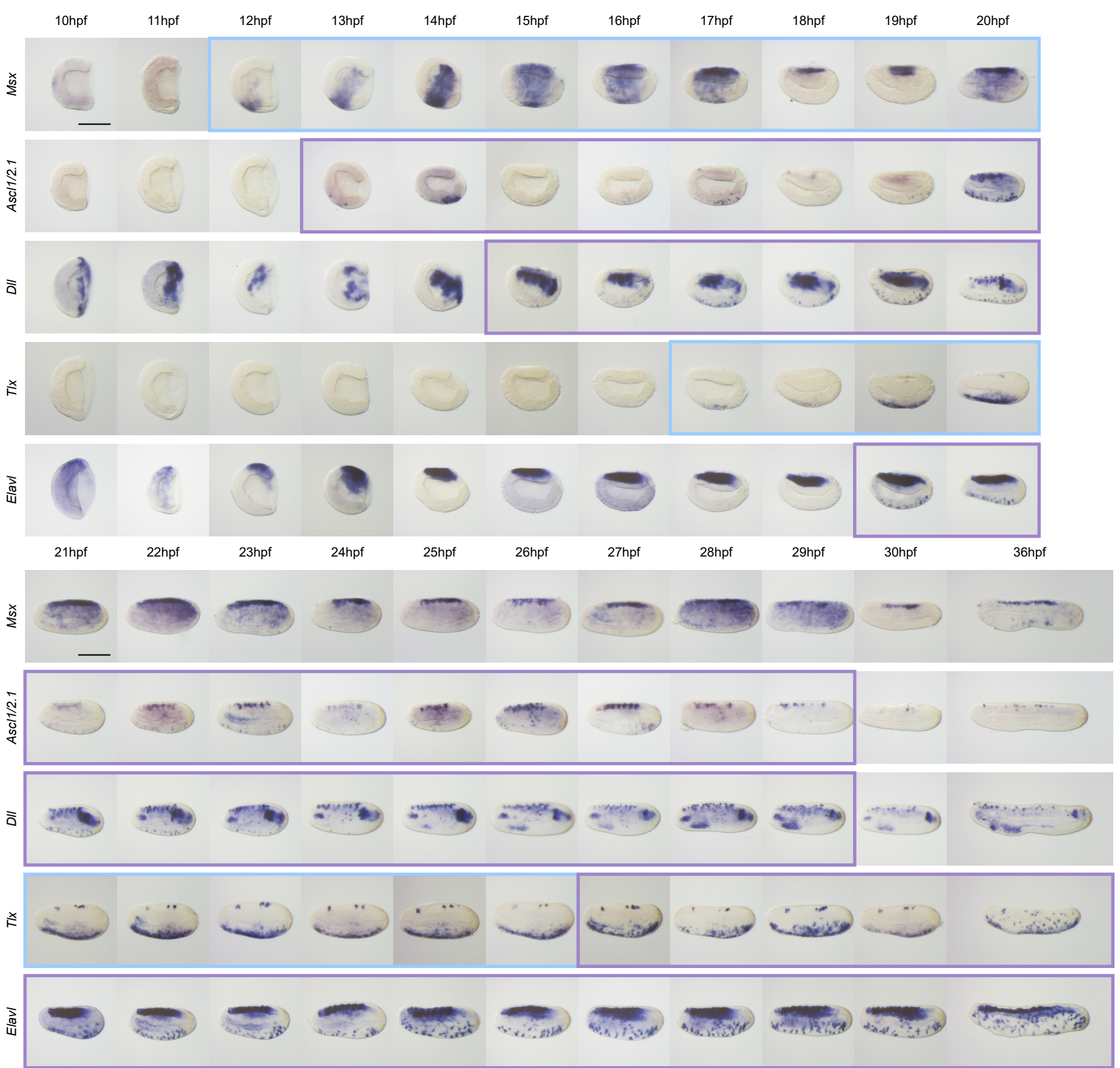

Supplement: Supplementary file 3 — Additional file 3: Fig. S3. Dynamical expression of vPNS genes in B. lanceolatum embryos. In situ hybridization at several stages from early gastrula to premouth larva for the B. lanceolatum vPNS genes: Msx, Ascl1/2.1, Dll, Tlx and Elavl. Light blue frame indicates expression in ventral neurogenic field and purple frame in ESNs. Embryos are shown in lateral view with dorsal to the top and anterior to the left. Scale bars: 50 μm. [file 12915_2022_1355_MOESM3_ESM.pdf]

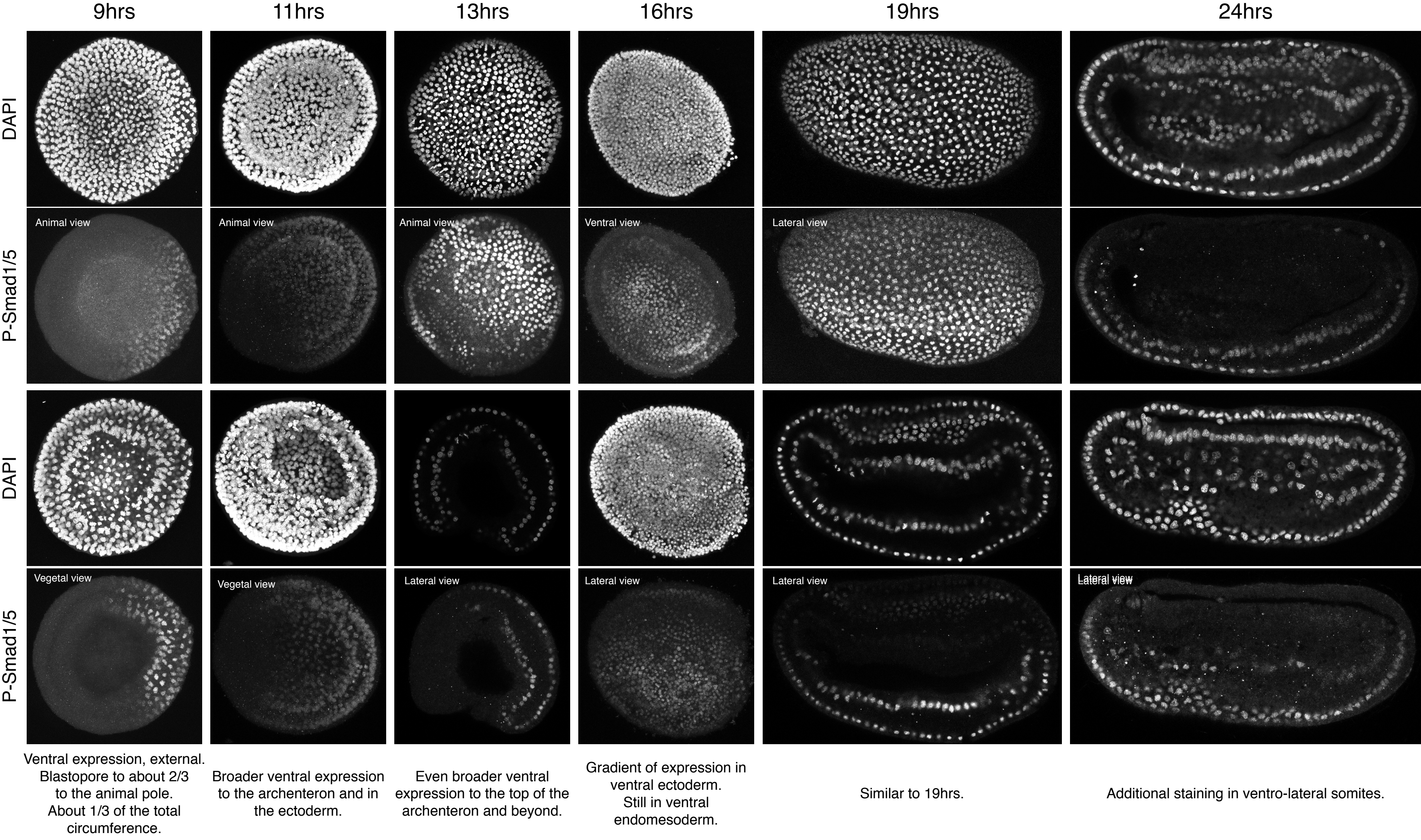

Supplement: Supplementary file 4 — Additional file 4: Fig. S4. BMP signaling activity during embryogenesis of amphioxus. BMP signaling was detected with an antibody against phosphorylated Smad1/5/8 in B. lanceolatum embryos from early gastrula stage (9 hpf) to late neurula stage (24 hpf). DAPI staining shows nuclei of the same embryo for which BMP signaling activity was detected. View of each embryo is indicated in top-left corner of each figure. [file 12915_2022_1355_MOESM4_ESM.pdf]

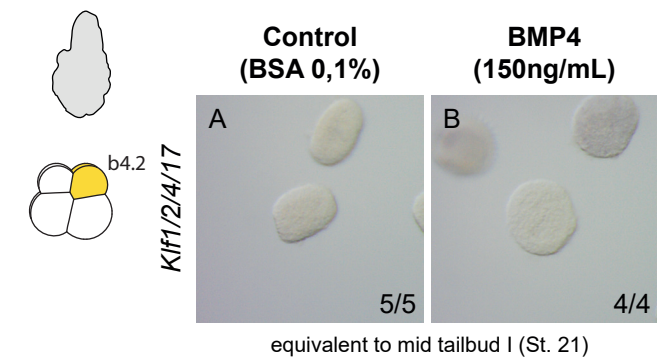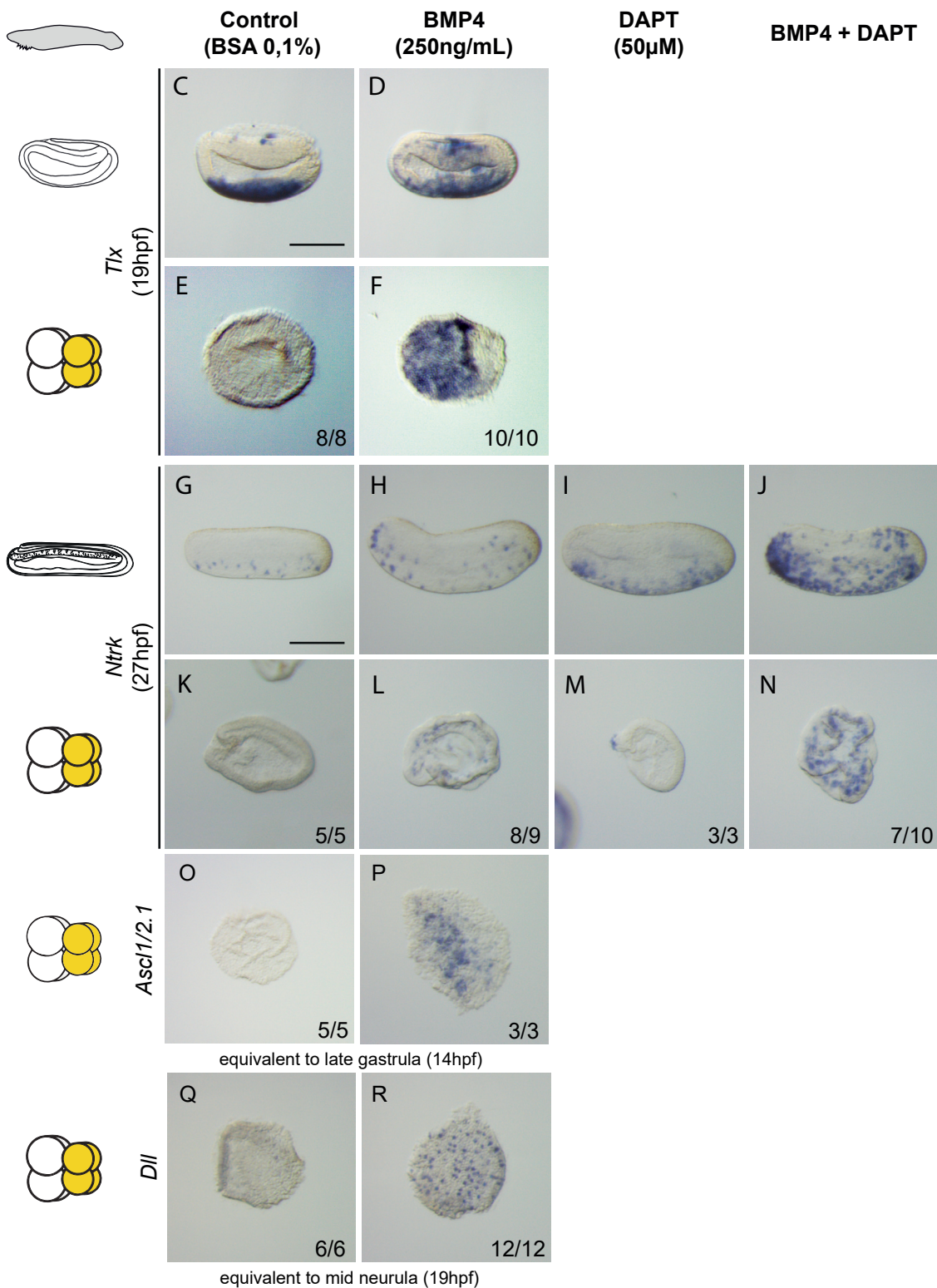

Supplement: Supplementary file 6 — Additional file 6: Fig. S6. Effects of BMP activation on ectodermal explants of P. mammillata and B. lanceolatum. Expression of P. mammillata midlines marker Klf1/2/4/17 (A, B) and B. lanceolatum ventral ectodermal marker Tlx (C-F) and ESNs markers Ntrk (G-N), Ascl1/2.1 (O, P) and Dll (Q, R) in whole embryos (C, D, G-J) and ectodermal explants (A, B, E, F, K-R) at several stages. In situ hybridization of vPNS genes in control embryos or ectodermal explants (A, C, E, G, K, O, Q), following treatment with BMP4 protein from 8-cell stages (B, D, F, H, L, P, R), with DAPT (I, M) or a combination of BMP4 and DAPT (J, N). The number of whole embryos analysed for each condition is between 15 to 40, and the number of explants is indicated in the bottom-right corner of the figure. All experiments have been done twice except for Ntrk and Ascl1/2.1 that have been done once. Scale bar: 50 μm. [file 12915_2022_1355_MOESM6_ESM.pdf]

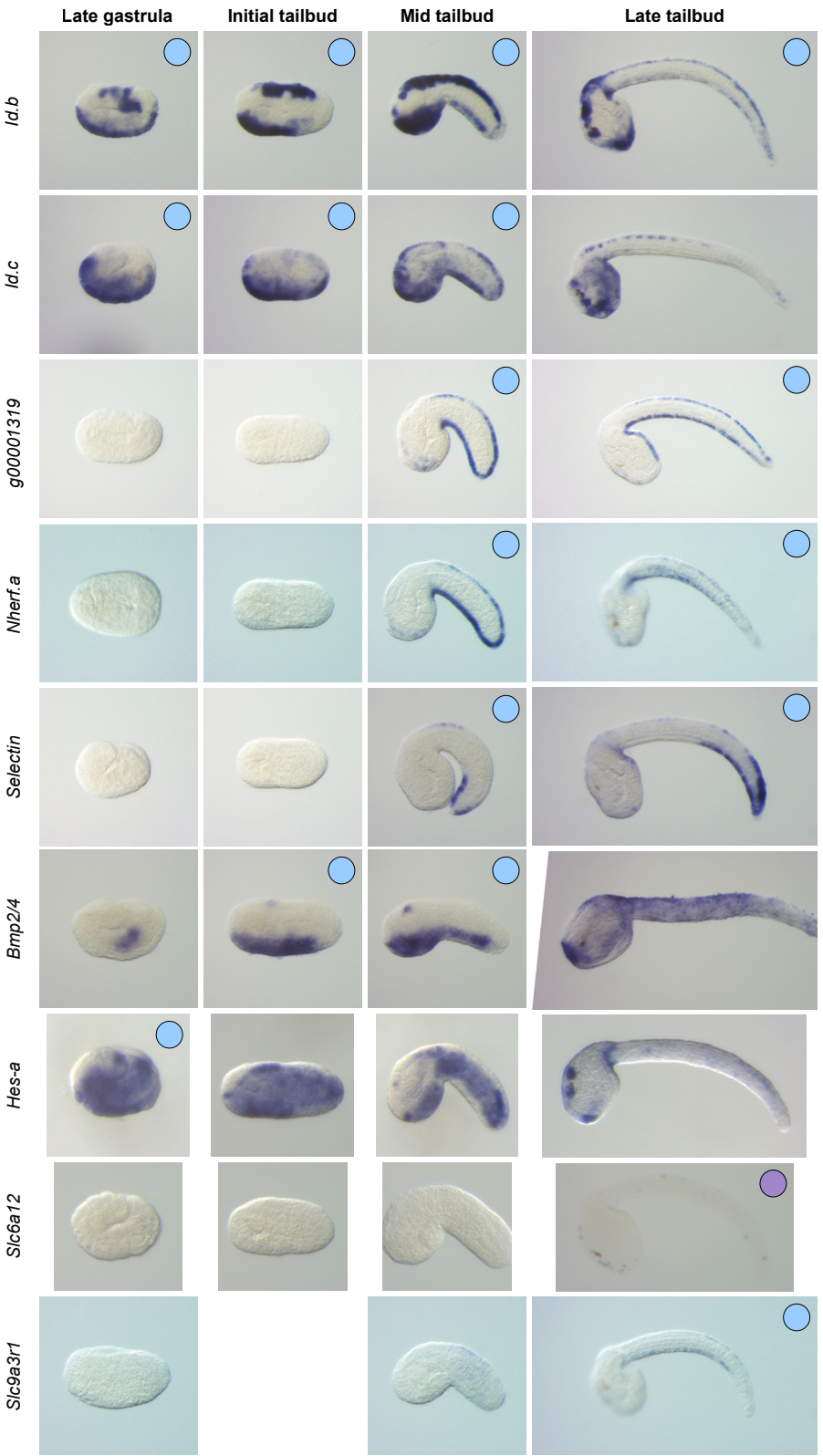

Supplement: Supplementary file 7 — Additional file 7: Fig. S7. Other candidate genes expressed in the vPNS of P. mammillata. In situ hybridization of other P. mammillata candidate genes at several stages with newly identified expression in vPNS. Genes expressed in the VML are represented by a blue circle and genes expressed in ventral ESNs by a purple circle. Embryos are shown in lateral view with dorsal to the top and anterior to the left. [file 12915_2022_1355_MOESM7_ESM.pdf]

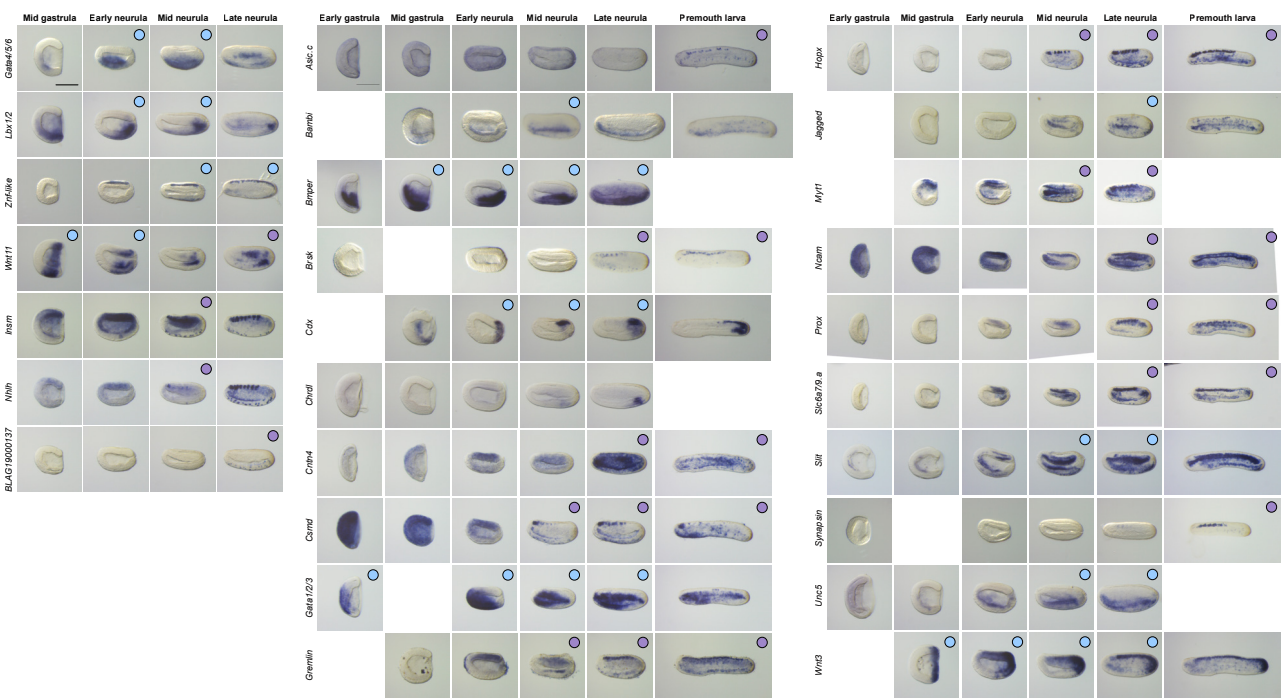

Supplement: Supplementary file 8 — Additional file 8: Fig. S8. Other candidate genes expressed in vPNS in B. lanceolatum. In situ hybridization of other B. lanceolatum candidate genes at several stages with newly identified expression in vPNS. Genes expressed in the VML are represented by a blue circle and genes expressed in ESNs by a purple circle. Embryos are shown in lateral view with dorsal to the top and anterior to the left. [file 12915_2022_1355_MOESM8_ESM.pdf]

**Control**  
**(DMSO 0,1%)**

**1-azakenpauillone**  
**at 12hpf (10 $\mu$ M)**

***Tlx***

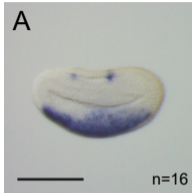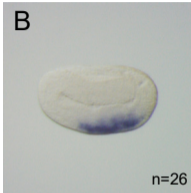

***Elavl***

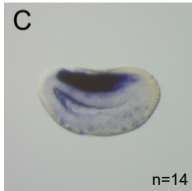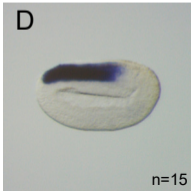

**Control**  
**(DMSO 0,1%)**

**C59**  
**at 12hpf (10 $\mu$ M)**

***Elavl***

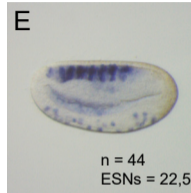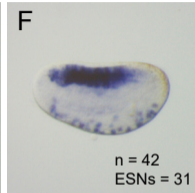

Supplement: Supplementary file 10 — Additional file 10: Fig. S10. Wnt signaling modulates vPNS genes expression in B. lanceolatum. (A-D) In situ hybridization for Tlx and Elavl at mid neurula stages in control embryos (A, C) or following activation of Wnt pathway from late gastrula stages (12 hpf) with 10 μM of 1-azakenpaullone (B, D). (E, F) In situ hybridization for Elavl at mid neurula stages in control embryos (E) or following inhibition of Wnt pathway from late gastrula stages (12 hpf) with 10 μM of C59 (F). Embryos are shown in lateral view with dorsal to the top and anterior to the left. The number of embryos analysed showing the displayed phenotype and, when counted, mean number of ESNs are indicated in the bottom-right corner of the figure. All experiments have been performed once. Scale bar: 50 μm. [file 12915_2022_1355_MOESM10_ESM.pdf]
