# Supplementary material for: Highly distinct genetic programs for peripheral nervous system formation in chordates
Source: BMC Biol. 2022 Jun 27;20:152. doi: 10.1186/s12915-022-01355-7 (PMC9238270; doi:10.1186/s12915-022-01355-7)
Supplement: Supplementary file 5 — Additional file 5: Fig. S5. Timing of vPNS sensitivity to BMP activation and inhibition in B. lanceolatum. In situ hybridization for Dll at neurula stages in control embryos (A, F), following continuous treatment with recombinant BMP4 protein starting at various stages (B-E), with dorsomorphin continuously starting at various stages (G-M) or for two hours at the stage indicated on the left of the figure before being extensively washed in seawater (N-S). Embryos are shown in lateral view with dorsal to the top and anterior to the left. Number of embryos analysed and the mean number of ESNs are indicated in the bottom-right corner of the figure. All experiments have been done once except those shown in B and E that have been done twice or more. Scale bar: 50 μm. [file 12915_2022_1355_MOESM5_ESM.pdf]

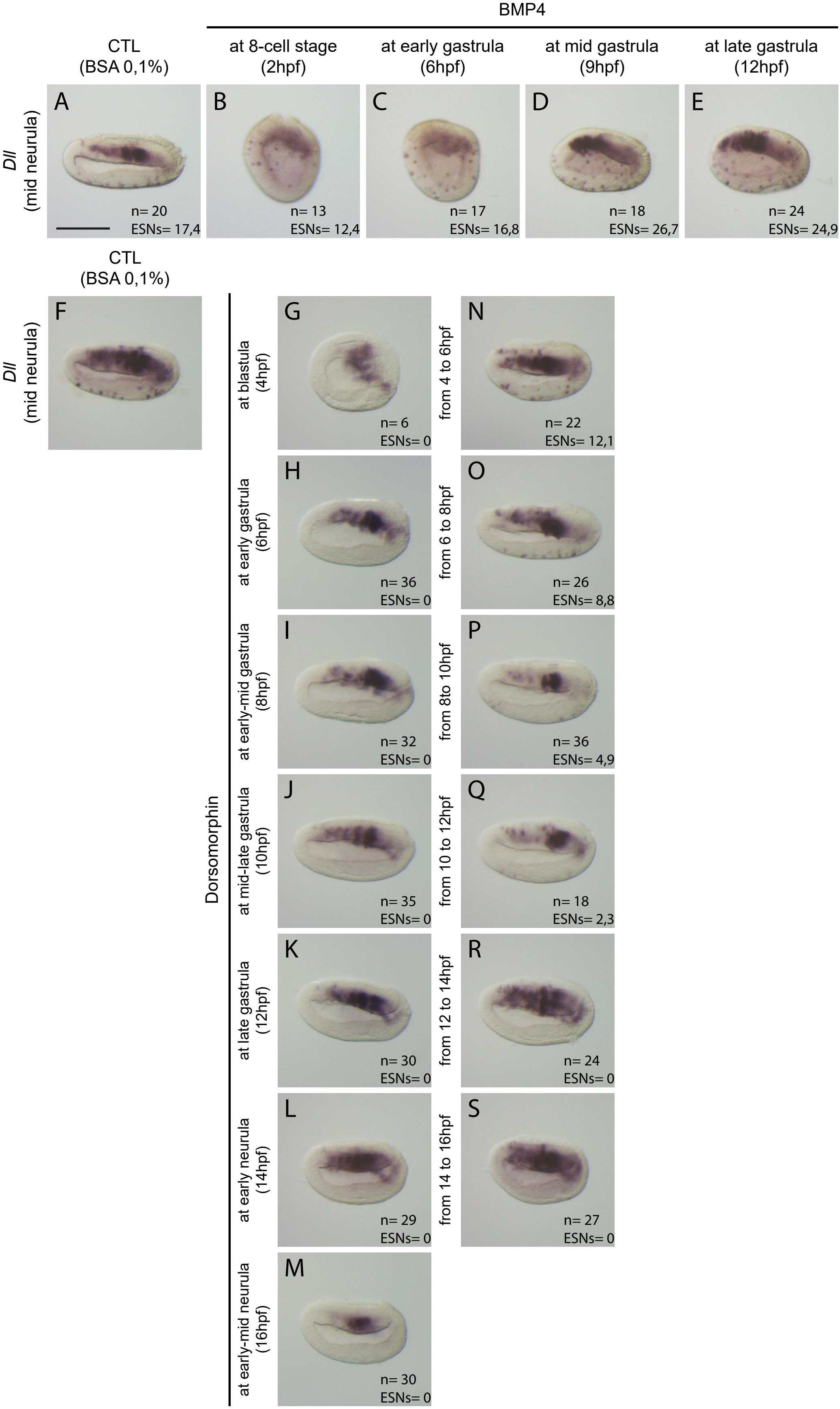
BMP4
CTL
(BSA 0,1%)
at 8-cell stage
(2hpf)
at early gastrula
(6hpf)
at mid gastrula
(9hpf)
at late gastrula
(12hpf)
Dll
(mid neurula)
A
n= 20
ESNs= 17,4
B
n= 13
ESNs= 12,4
C
n= 17
ESNs= 16,8
D
n= 18
ESNs= 26,7
E
n= 24
ESNs= 24,9
CTL
(BSA 0,1%)
F
Dll
(mid neurula)
Dorsomorphin
at blastula
(4hpf)
G
n= 6
ESNs= 0
from 4 to 6hpf
N
n= 22
ESNs= 12,1
at early gastrula
(6hpf)
H
n= 36
ESNs= 0
from 6 to 8hpf
O
n= 26
ESNs= 8,8
at early-mid gastrula
(8hpf)
I
n= 32
ESNs= 0
from 8to 10hpf
P
n= 36
ESNs= 4,9
at mid-late gastrula
(10hpf)
J
n= 35
ESNs= 0
from 10 to 12hpf
Q
n= 18
ESNs= 2,3
at late gastrula
(12hpf)
K
n= 30
ESNs= 0
from 12 to 14hpf
R
n= 24
ESNs= 0
at early neurula
(14hpf)
L
n= 29
ESNs= 0
from 14 to 16hpf
S
n= 27
ESNs= 0
at early-mid neurula
(16hpf)
M
n= 30
ESNs= 0
