# Supplementary material for: Highly distinct genetic programs for peripheral nervous system formation in chordates
Source: BMC Biol. 2022 Jun 27;20:152. doi: 10.1186/s12915-022-01355-7 (PMC9238270; doi:10.1186/s12915-022-01355-7)
Supplement: Supplementary file 9 — Additional file 9: Fig. S9. Effects of modulating BMP and Notch signaling pathways on new B. lanceolatum vPNS genes. (A-O) In situ hybridization for other vPNS candidate genes at early neurula (A-I) or mid neurula (J-O) stages in control embryos (A, D, G, J, M), following treatment from late gastrula stages with BMP4 protein (B, E, H, K, N) or dorsomorphin (C, F, I, L, O). (P-A’) In situ hybridization for other vPNS candidate genes at mid (P-W) or late neurula (X-A’) stages in control embryos (P, T, X), following treatment from late gastrula stages with BMP4 protein (Q, U, Y), DAPT (R, V, Z) or combined treatment with BMP4 and DAPT (S, W, A’). Black arrowheads indicate ESN when not clearly visible and arrows indicate the anterior most epidermis of the embryo presenting an accumulation of ectopic ESNs. Embryos are shown in lateral view with dorsal to the top and anterior to the left. Each experiment has been performed twice. Scale bars: 50 μm. [file 12915_2022_1355_MOESM9_ESM.pdf]

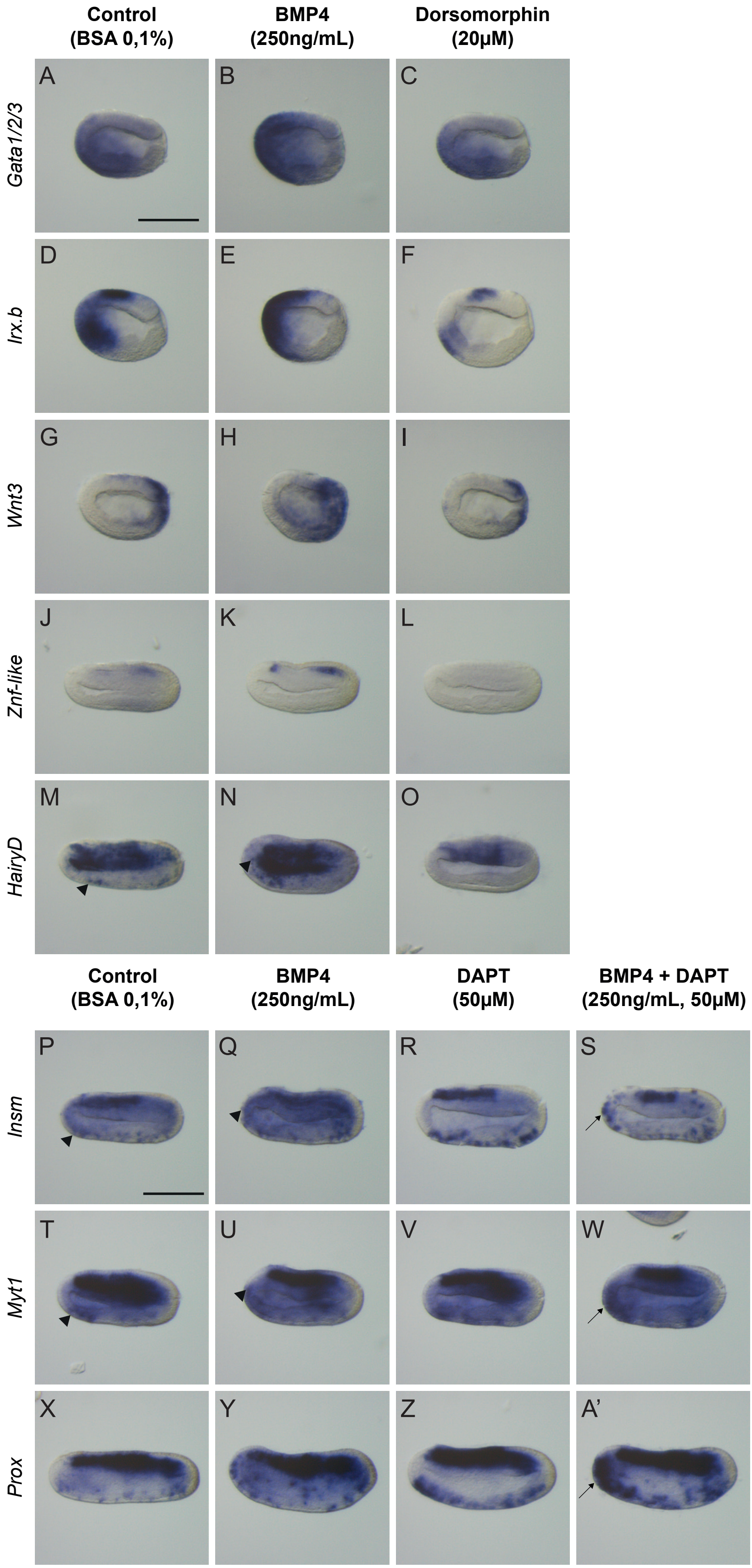
Control (BSA 0,1%)
BMP4 (250ng/mL)
Dorsomorphin (20µM)
Gata1/2/3
Irx.b
Wnt3
Znf-like
HairyD
Control (BSA 0,1%)
BMP4 (250ng/mL)
DAPT (50µM)
BMP4 + DAPT (250ng/mL, 50µM)
Insm
Myt1
Prox
A
B
C
D
E
F
G
H
I
J
K
L
M
N
O
P
Q
R
S
T
U
V
W
X
Y
Z
A'
