# Supplementary material for: Highly distinct genetic programs for peripheral nervous system formation in chordates
Source: BMC Biol. 2022 Jun 27;20:152. doi: 10.1186/s12915-022-01355-7 (PMC9238270; doi:10.1186/s12915-022-01355-7)
Supplement: Supplementary file 11 — Additional file 11: Fig. S11. Invertebrate chordates vPNS genes are expressed in PNS territories in mouse. Expression of mouse orthologs were obtained with MGI database (http://www.informatics.jax.org) for all invertebrate chordates vPNS genes (grey), ventral epidermis genes of amphioxus (light blue) and ascidians (dark blue), and ESN of amphioxus (light purple) and ascidians (dark purple). (A) Expression of orthologs of invertebrate chordates vPNS genes in several mouse embryonic territories. (B) Proportion of mouse orthologs of vPNS genes among the genes expressed in different territories. [file 12915_2022_1355_MOESM11_ESM.pdf]

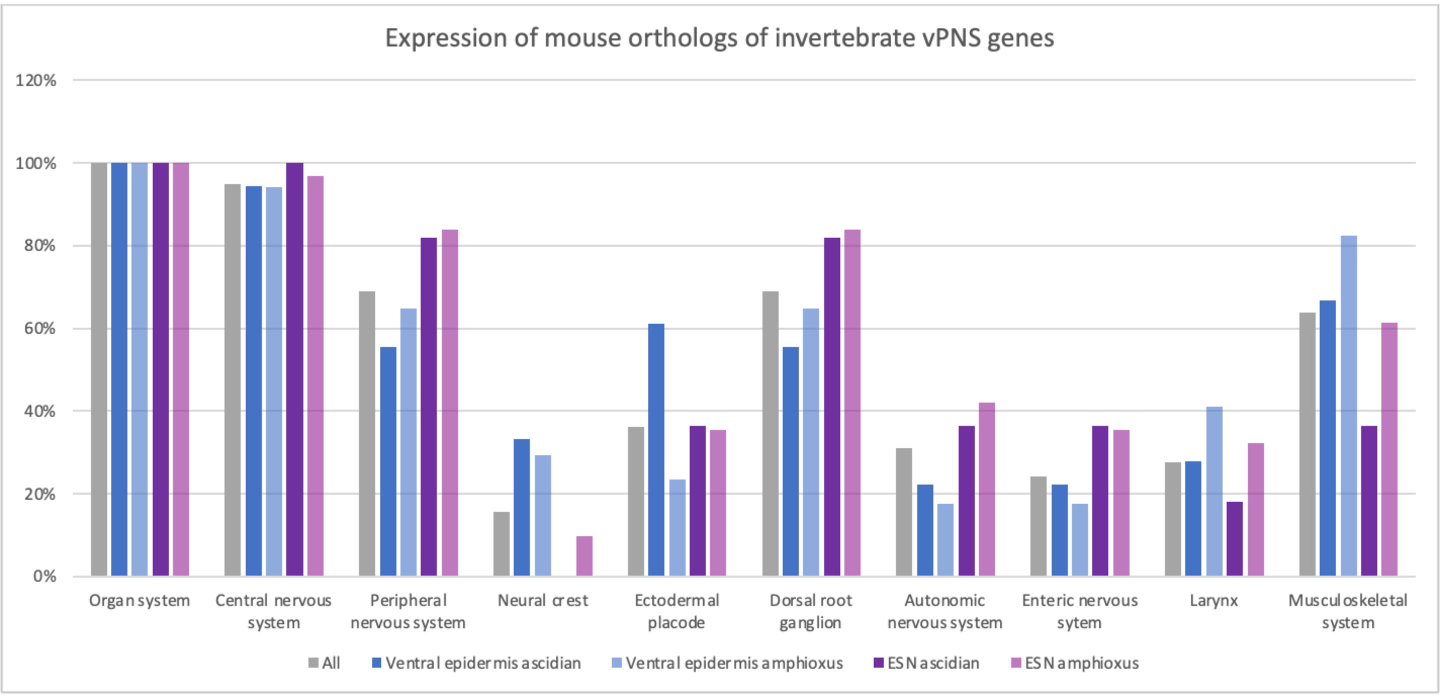
Expression of mouse orthologs of invertebrate vPNS genes
120%
100%
80%
60%
40%
20%
0%
Organ system
Central nervous system
Peripheral nervous system
Neural crest
Ectodermal placode
Dorsal root ganglion
Autonomic nervous system
Enteric nervous sytem
Larynx
Musculoskeletal system
All
Ventral epidermis ascidian
Ventral epidermis amphioxus
ESN ascidian
ESN amphioxus

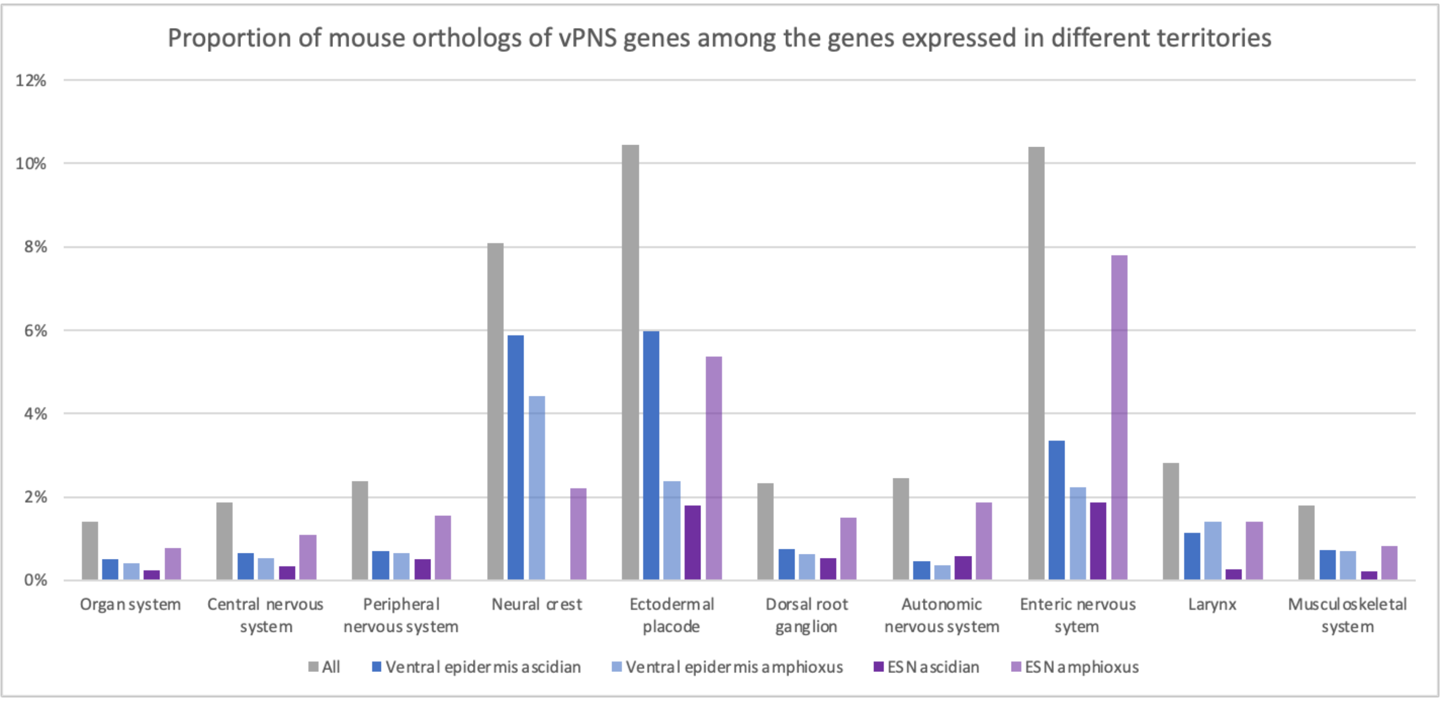
Proportion of mouse orthologs of vPNS genes among the genes expressed in different territories
12%
10%
8%
6%
4%
2%
0%
Organ system
Central nervous system
Peripheral nervous system
Neural crest
Ectodermal placode
Dorsal root ganglion
Autonomic nervous system
Enteric nervous sytem
Larynx
Musculoskeletal system
All
Ventral epidermis ascidian
Ventral epidermis amphioxus
ESN ascidian
ESN amphioxus
